# Development process of a consensus-driven CONSORT extension for randomised trials using an adaptive design

**DOI:** 10.1186/s12916-018-1196-2

**Published:** 2018-11-16

**Authors:** Munyaradzi Dimairo, Elizabeth Coates, Philip Pallmann, Susan Todd, Steven A. Julious, Thomas Jaki, James Wason, Adrian P. Mander, Christopher J. Weir, Franz Koenig, Marc K. Walton, Katie Biggs, Jon Nicholl, Toshimitsu Hamasaki, Michael A. Proschan, John A. Scott, Yuki Ando, Daniel Hind, Douglas G. Altman

**Affiliations:** 1 School of Health and Related Research, University of Sheffield, Regent Court, 30 Regent Street, Sheffield, S1 4DA UK; 2 Centre for Trials Research, Cardiff University, Cardiff, UK; 3 University of Reading, Reading, UK; 4 Lancaster University, Lancaster, UK; 5 MRC Biostatistics Unit, University of Cambridge, Cambridge, UK; 6 University of Edinburgh, Edinburgh, UK; 7 Centre for Medical Statistics, Informatics, and Intelligent Systems, Medical University of Vienna, Vienna, Austria; 8 Janssen Pharmaceuticals, Titusville, New Jersey, USA; 9 National Cerebral and Cardiovascular Center, Suita, Japan; 10 National Institute of Allergy and Infectious Diseases, National Institutes of Health, Bethesda, USA; 11 Division of Biostatistics in the Center for Biologics Evaluation and Research, Food and Drug Administration, White Oak, USA; 12 Pharmaceuticals and Medical Devices Agency, Tokyo, Japan; 13 University of Oxford, Oxford, UK; 14 Institute of Health and Society, Newcastle University, Newcastle upon Tyne, UK

**Keywords:** Adaptive design, Flexible design, CONSORT extension, Reporting guidance, Reporting guideline, Randomised controlled trial

## Abstract

**Background:**

Adequate reporting of adaptive designs (ADs) maximises their potential benefits in the conduct of clinical trials. Transparent reporting can help address some obstacles and concerns relating to the use of ADs. Currently, there are deficiencies in the reporting of AD trials. To overcome this, we have developed a consensus-driven extension to the CONSORT statement for randomised trials using an AD. This paper describes the processes and methods used to develop this extension rather than detailed explanation of the guideline.

**Methods:**

We developed the guideline in seven overlapping stages:

1. Building on prior research to inform the need for a guideline;
2. A scoping literature review to inform future stages;
3. Drafting the first checklist version involving an External Expert Panel;
4. A two-round Delphi process involving international, multidisciplinary, and cross-sector key stakeholders;
5. A consensus meeting to advise which reporting items to retain through voting, and to discuss the structure of what to include in the supporting explanation and elaboration (E&E) document;
6. Refining and finalising the checklist; and
7. Writing-up and dissemination of the E&E document.

The CONSORT Executive Group oversaw the entire development process.

**Results:**

Delphi survey response rates were 94/143 (66%), 114/156 (73%), and 79/143 (55%) in rounds 1, 2, and across both rounds, respectively. Twenty-seven delegates from Europe, the USA, and Asia attended the consensus meeting. The main checklist has seven new and nine modified items and six unchanged items with expanded E&E text to clarify further considerations for ADs. The abstract checklist has one new and one modified item together with an unchanged item with expanded E&E text. The E&E document will describe the scope of the guideline, the definition of an AD, and some types of ADs and trial adaptations and explain each reporting item in detail including case studies.

**Conclusions:**

We hope that making the development processes, methods, and all supporting information that aided decision-making transparent will enhance the acceptability and quick uptake of the guideline. This will also help other groups when developing similar CONSORT extensions. The guideline is applicable to all randomised trials with an AD and contains minimum reporting requirements.

**Electronic supplementary material:**

The online version of this article (10.1186/s12916-018-1196-2) contains supplementary material, which is available to authorized users.

## Introduction

Clinical trials are expected to adhere to high ethical and scientific standards and answer research questions robustly, as quickly as possible to benefit patients, and use no more research resources than necessary. The need to streamline the conduct of trials is a cross-sector (public and private sector) and regulatory priority [1–6]. Well-designed and properly conducted adaptive design (AD) trials can improve the efficiency of clinical trials and help achieve these objectives.

There is a growing interest in ADs across sectors to address the shortcomings of trials with a fixed design. Furthermore, there is considerable statistical methodological literature on ADs [7, 8] and new methods continue to be developed. Discussions on opportunities to use ADs across trial phases and advice on their robust design and conduct are growing [9–17]. Different types of ADs are increasingly used or at least considered at the design stage across sectors [18–25]. However, ADs have a number of issues and challenges. There is lack of practical knowledge of ADs, and some obstacles and concerns about some types of ADs are impeding their use [22, 26–32]. Access to case studies of AD trials may help alleviate some of these problems [28, 33]. Consequently, authors have reviewed real-life AD case studies to build knowledge resources [18, 19, 34, 35]. Although these reviews found a number of AD case studies, especially in oncology, many of these trials are inadequately reported and thus may not address some of the concerns about ADs [18, 33, 36]. Adequate reporting will improve the credibility and interpretability of ADs and increase their application [28, 34].

The Consolidated Standards of Reporting Trials (CONSORT) framework has been instrumental in promoting transparent reporting of randomised trials. Increased complexity of the trial design and conduct, as is common in AD trials, comes with additional transparency and reporting demands. The CONSORT 2010 statement [37] includes the concept of changes to the trial design and methods after commencement without differentiating between planned adaptations and unplanned changes (item 3b) and interim stopping rules (item 7b). It does not, however, specifically address the general reporting needs for randomised trials that use an AD. As noted above, reporting deficiencies of AD trials have been highlighted [18, 23, 33–35] and it has been suggested that there is a need for additional reporting considerations to address this [33–35, 38]. However, these papers lack a grounded methodological approach to developing comprehensive reporting guidance. Thus, the suggested piecemeal recommendations are likely to be incomplete and unlikely to be accepted to influence practice because they lack input from important stakeholders through a robust process. Therefore, this project aimed to address this limitation by using a recommended consensus-driven framework [39] to develop an official reporting guideline, Adaptive designs CONSORT Extension (ACE), for randomised trials that use ADs.

In the spirit of good reporting practice, this paper describes the processes and methods that the ACE Steering Committee (SC) used to develop a consensus-driven ACE reporting guideline. We provide justification for the decisions made to arrive at the final checklist and explain the structure of the forthcoming ACE explanation and elaboration (E&E) document. Box 1 lists the long-term objectives of the ACE project.

## Methods

**Fig. 1:**
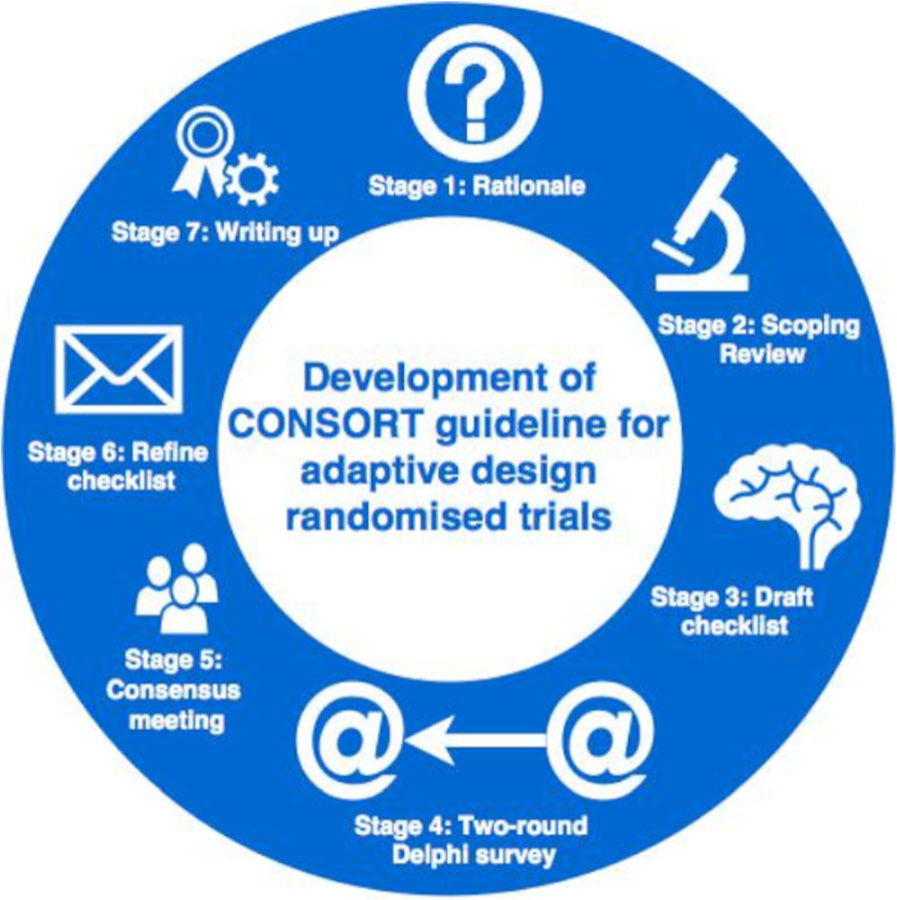
Development process of the Adaptive designs Extension CONSORT extension guideline for randomised trials

A favourable ethical approval for this study was granted by the Research Ethics Committee (REC) of the School of Health and Related Research (ScHARR) at the University of Sheffield (ref: 012041). The guideline development process adhered to a consensus-driven methodological framework for developing healthcare reporting guidelines recommended by the CONSORT Executive Group [39]. An a priori registered protocol accessible via the EQUATOR Network [40] guided the conduct of this research, and Fig. 1 summarises the development process.

### Study management and group composition

A multidisciplinary SC of 19 members from industry and the public sector, including the CONSORT Executive Group representative (DA) and members of the MRC Network of Hubs for Trials Methodology Research (HTMR) Adaptive Designs Working Group (ADWG), led the guideline development process. The members were based in Europe, USA, and Asia. The professional experience of members included methodology and conduct of AD trials, management and conduct of randomised trials, regulatory assessment and approval, reviewing research grant applications and decision-making on research funding panels, systematic reviewing of evidence, and development of reporting guidelines. This composition was motivated by the need to capture diverse views of experts across sectors with multidisciplinary roles in trials research covering wide geographical locations.

A Study Management Group (SMG) comprised of thirteen SC members oversaw the day-to-day project activities in consultation with the SC. For quality control, we sought the advice from an External Expert Panel of four members based in the USA, UK, and Australia—with practical and methodological expertise in AD trials during the drafting process of the version of the checklist to be included in the Delphi surveys. Additional file 1 summarises the project activities undertaken throughout the development process.

### Prior work to inform the need for a CONSORT extension

The findings from a National Institute for Health Research (NIHR) Doctoral Research Fellowship (DRF-2012-05-182) led by MD and supervised by SJ, ST, and JN informed the need for this research [33]. The idea was presented, discussed, and contextualised at the 2016 annual workshop of the MRC HTMR ADWG attended by six members of the ACE SC (MD, TJ, PP, JW, AM, and CW). In summary, research prior to 2016 investigated obstacles and potential facilitators to the use of AD trials [22, 26, 28–32, 41] as well as deficiencies in their reporting [18, 23, 33, 34]. Further research highlighted the overwhelming need for a tailored reporting guideline for AD trials with literature suggesting some reporting principles [26, 28, 33–35, 38].

We approached the CONSORT Executive Group in 2016 informing them about our plans for the ACE guideline, and they agreed to oversee the development process. Before the research began, we further performed a scoping free text search on 10 October 2016 using the term ‘adaptive’ on the EQUATOR Network database [42], but we found no reporting guideline on ADs or related guideline under development.

### Scoping literature review

The objectives of the scoping narrative review were to collate any concerns about AD trials or considerations that may influence their reporting, to identify any suggestions on how AD trials should be reported and to establish definitions of technical terms. The aim was to guide the preliminary drafting of the reporting items and working definitions for the extension checklist. The review also helped us to create a list of authors who had published AD trials or methodology research as potential participants for the Delphi surveys.

The literature search was not intended to be exhaustive but to provide a good foundation for the guidance development process. We searched the MEDLINE database via PubMed on 17 November 2016 for any articles about randomised AD trials written in English using this combination of terms: ((“adaptive design”) OR (“adaptive clinical trial”) OR (“adaptive trial”) OR (“adaptive interim”) OR (“flexible design”)) AND (reporting OR recommendation* OR (“best practice”) OR (“good practice”) OR (“panel discussion*”) OR guidance OR guideline* OR interpretation OR bias OR (“expert opinion”) OR (“expert panel”)). We retrieved 237 articles, from which we excluded 51: 33 were ineligible (irrelevant to the subject or about non-randomised studies), 16 inaccessible, one duplicate, and one had an English abstract but was written in Chinese. We narratively reviewed 186 eligible publications, and key ones are cited in relevant sections. We also reviewed some additional key documents that we were aware of but that were not retrieved by the search strategy, such as regulatory reflection guidance [4–6]. We summarised the findings and drafted a preliminary checklist in preparation for our first face-to-face SC meeting.

### Checklist drafting process

On 29 January 2017, the SC met in Sheffield for a full day to discuss the findings from the scoping review, agree upon a working definition of an AD trial, and to discuss the preliminary extension checklist in the context of the concerns about AD trials and what necessary changes should be made to the CONSORT 2010 checklist.

#### What do we consider an adaptive design trial?

We found several references that provide definitions of an AD and related technical terms [5, 6, 16, 43–46]. Our review showed that what is considered an AD trial is inconsistently defined and often creates confusion [26, 41, 43]. However, there are three common themes in the definitions [5, 6, 16, 43, 46]: ‘use of accruing trial data’, ‘opportunity to make changes to aspects of the trial’, and ‘need to preserve trial validity and integrity’. After a lengthy discussion, the SC agreed to define an AD as:

A clinical trial design that offers pre-planned opportunities to use accumulating trial data to modify aspects of an ongoing trial while preserving the validity and integrity of that trial

By *pre-planned*, we envisaged trial changes or adaptations are specified at the design stage or at least before any unblinded review of the accumulating trial data, and they are documented in an auditable trial-related document such as the trial protocol. We acknowledged the existence of flexible statistical methods to cope with unplanned trial changes under specific conditions [7]. However, we strongly feel that pre-planning is one of the necessary conditions to preserve the integrity of the trial, a view shared with regulatory guidance [4–6]. Thus, this guideline is not meant for trials with unplanned changes only (no planned adaptations).

For the scope of this guideline, changes to *aspects of an ongoing trial* that solely depend on external information rather than *accumulating trial data* are outside the scope of what we consider an AD trial. Furthermore, we specifically exclude the use of accruing trial data to make changes that relate only to the feasibility and process aspects of conducting a trial, which forms part of almost every trial. We refer to these changes as operational adaptations [47]. The types of ADs and trial aspects that can be modified are discussed in the literature [3, 9, 11, 15, 16, 24, 41, 48–53].

By *validity*, we meant the ability to provide correct statistical inference to establish the effects of the study interventions and produce accurate estimates of the effects (such as point estimates and associated uncertainty) to give results that are convincing to research consumers. Finally, the use of the word *integrity* pertains to minimisation of operational bias, maintenance of data confidentiality, and consistency in trial conduct for credibility, interpretability, and persuasiveness of trial results. Our definitions of terms relating to ADs are listed in Additional file 2.

#### What are the concerns for adaptive design trials?

The review found some key publications that discussed why the reporting of AD trials requires special consideration and reporting suggestions or recommendations for particular types of AD trials [23, 25, 33, 34, 38, 45, 51, 53–61]. ADs are not immune to potential biases and limitations despite their appealing nature and promising benefits [9, 50, 53].

Box 2 summarises the concerns or considerations that influence the reporting of ADs into eight themes that may depend on the type of the AD and scope of the trial adaptations used. These themes explain why the reporting of AD trials requires special consideration, and they influenced the development of the ACE guideline.

#### Drafting of the first extension checklist

The SC then discussed the preliminary extension checklist drafted during the scoping literature review focusing on what changes need to be made and the structure of the changes with justification. We classified items as ‘no changes proposed’, ‘modifications proposed’, and ‘new item suggested’. A report summarising the discussions is accessible online (see download at 10.15131/shef.data.6139631). Following the first face-to-face meeting in Sheffield, the checklist was then redrafted and refined during an iterative process through subsequent face-to-face and teleconference meetings and email correspondence involving the SMG and the SC.

The External Expert Panel reviewed the draft checklist and working definitions of technical terms. We added two specific items on how to deal with overrunning participants (12e) and multiple outcomes or multiple treatment comparions (12f), which were suggested by the panel (see download at 10.15131/shef.data.6198290). The panel also suggested a rewording of some items for clarification and identified specific aspects that should be addressed in the E&E document. In addition, independent experts were consulted to review the draft checklist to identify major problems with content and wording of items.

On 5 May 2017, the SC finalised the official first draft of the extension checklist with a total of 58 items. This list included 22 new items, 15 modified items, and 21 items unchanged from the CONSORT 2010 checklist. This draft checklist is accessible online (see download at 10.15131/shef.data.6198290).

### The sampling frame for the Delphi surveys

We aimed to engage key stakeholders across sectors and over wide geographical locations. We targeted those with AD-related experience including clinical trialists, clinical investigators, statisticians, trial methodologists, and health economists; those interested in using ADs; and consumers of research findings, decision makers, and policy-makers in clinical trials research including journal editors, systematic reviewers, research funders, regulators, research ethicists, and patient representative groups.

We created a list of 468 authors of the AD-related publications (trials or methodology) from our review and known case studies [18, 34]. This list contributed to the majority of the survey sampling frame. The details of organisations or professional groups we also approached are accessible online (see download at 10.15131/shef.data.6291050). We used a wide range of platforms to reach out to key stakeholders of interest such as targeted mailing lists, social media, and personal communications (see Additional file 3).

### The Delphi process

The National Perinatal Epidemiology Unit (NPEU, University of Oxford) built and hosted the online Delphi surveys and offered administrative support to maintain the anonymity of participants’ responses. The SC including the lead investigator and study coordinator did not have access to any information that could link participants to their responses during and after the survey.

#### Number of survey rounds

The objective of the Delphi process was to assess the stability of opinions that can be viewed as consistency in ratings of importance between rounds and not merely to reach consensus. We expected two survey rounds would suffice to reach stability in perceptions based on recent similar studies [62, 63]. However, the methodology permitted the SC the flexibility to undertake a third round if necessary based on the results and feedback received in round 2.

#### Scoring system

**Fig. 2:**
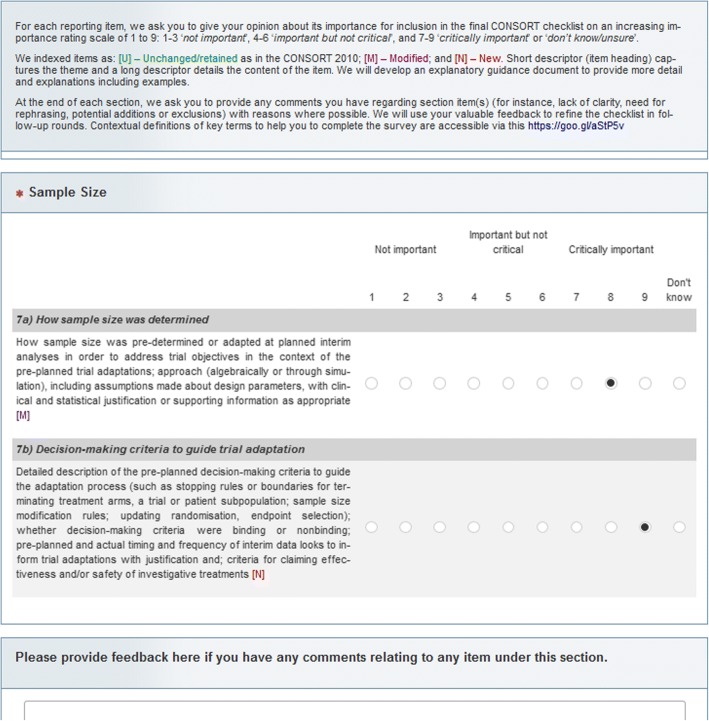
Snapshot of the online round 1 Delphi survey. [N] and [M] represent new and modified reporting items

We used an importance rating scale of 0 to 9 adopted in related Delphi surveys [62–64]: ‘not important’ (score 1 to 3), ‘important but not critical’ (score 4 to 6), ‘critically important’ (score 7 to 9), and ‘do not know’ (unsure). We used the same scoring system across rounds and indicated whether items were new (N), modified (M), or remained unchanged (U) from the CONSORT 2010 checklist [65]. See Fig. 2 for a screenshot.

#### Delphi round 1

We registered stakeholders who were willing to take part with informed consent via a bespoke web-based platform. During registration, we obtained informed consent and gathered demographics and characteristics of participants such as geographical location, self-identified stakeholder group (clinical trials user, clinical trialist, or methodologist), employment sector, years of experience in trials research, and AD-related research experience.

Registered participants were sent personalised emails with a link to the round 1 survey. The landing survey page stated the ACE project aims, the contextual definition of an AD trial, and the scope of the guidance. We asked participants to rate their perceptions about the importance of the suggested reported items. Unchanged items were included to allow participants to provide comments and assess completeness of the proposed extension checklist when completing the survey. We provided participants with the opportunity to give item-specific and general open-ended feedback such as any potentially overlooked modifications or clarity issues. We activated the round 1 survey on 31 May 2017 and gave participants approximately 3 weeks to complete it.

#### Delphi round 2

**Fig. 3:**
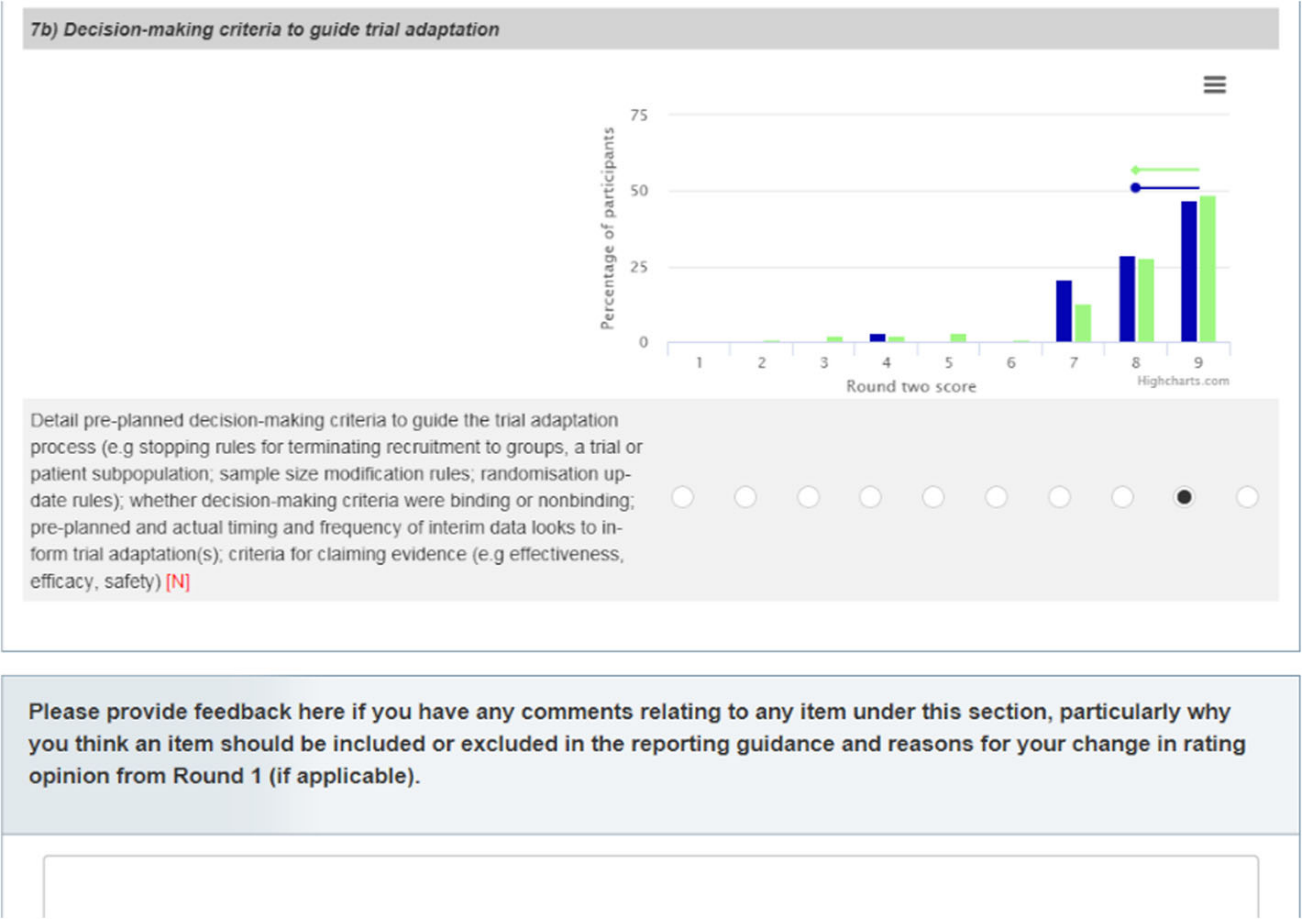
Snapshot of the online Delphi survey for round 2 among round 1 completers. In green are responses of all participants. In blue are the responses of the self-identified stakeholder group at registration which the participant belongs to (clinical trialist, clinical trial user, or methodologist)

Between rounds 1 and 2, we re-opened registration and extended recruitment to specifically target journal editors using a similar process as described for round 1. All registered participants were eligible to complete round 2 unless they withdrew consent. In round 2, participants who completed the round 1 survey were presented with their own previous item rating scores and the distribution of the item rating as displayed in Fig. 3 (including medians and interquartile ranges (IQRs) of all participants (green) and their self-identified stakeholder group at registration (blue)). We did not display previous data for participants who only completed the round 2 survey. We asked participants to rate the importance of 38 new or modified items as compared to the CONSORT 2010 checklist. Item 21 (generalisability) from round 1 was unintentionally overlooked and not included in the round 2 survey due to a technical error (see download at 10.15131/shef.data.6198290). Items 14a (dates defining the periods of recruitment) and 14b (unexpected termination/why the trial ended or stopped) were modified for reasons stated in Additional file 4. We asked participants to give open-ended feedback including any reasons for changing their ratings where applicable. The survey also displayed unchanged items from the CONSORT 2010 checklist and asked participants to provide any additional feedback without rating these items. The main and abstract draft checklist used for round 2 are accessible online (see download at 10.15131/shef.data.6198347). We launched the round 2 Delphi survey on 15 September 2017 and gave participants approximately 4 weeks to complete it.

#### Consensus decision-making criteria

We predefined consensus as receiving the support of at least 70% of responders rating an item as ‘critically important’ for inclusion in the round 2 Delphi survey [40, 66]. Prior to the consensus meeting, we specified that the decision to retain an item should be based on achieving at least 50% support of delegates voting to ‘keep’ an item [40]. These criteria in consideration with the feedback gathered informed the SC in making the final decisions about reporting items to be included in the ACE guideline.

### Analysis methods

We summarised the distribution of characteristics and demographics of registered participants and responders for each Delphi round. Item rating scores were descriptively analysed using the number of responders, the median (IQR), and mean (standard deviation, SD). We explored whether the ratings of participants differed by specific characteristics of interest using clustered boxplots stratified by:

- Self-selected key stakeholder group (clinical trial user, clinical trialist, or methodologist);
- Current employment sector (public sector or industry);
- Self-reported regulatory assessment experience (yes or no); and
- Primary role in clinical trials research as a statistician (yes or no).

We summarised the number and proportion of participants who rated an item as ‘not important’, ‘important but not critical’, and ‘critically important’, including the ‘do not know’ category. We analysed qualitative feedback gathered during the Delphi surveys using a simple thematic analysis [67] to identify common comments and elucidate feedback on suggested items (new or modified) as well as gather additional content suggestions for the checklist.

We assessed the stability and consistency of individual ratings of item importance across rounds using:

1. Percentage agreement as assessed by the proportion of responders whose ratings were the same in both rounds;
2. Weighted Cohen’s kappa with absolute error weights [68] with confidence intervals calculated using bootstrapping [69];
3. Bland-Altman plots [70] and histograms of changes in the scores between rounds.

### Decision-making process

#### Feedback-based adaptation process

The SC reviewed the open-ended feedback received to inform the development process, such as modification of items for clarification and testing the wording of items. For instance, in round 1, we tested the preference of two additional versions of item 14c adaptation decisions (14d pre-planned adaptation decisions and 14e deviations from pre-planned adaptation decisions, see download at 10.15131/shef.data.6198290). The wording of items and structuring of the checklist evolved during the process.

#### Consensus meeting and onwards

The aim of the consensus meeting was to discuss the round 2 Delphi survey results; to make advisory decisions on items to retain in the guideline through voting, including reasons for supporting decisions; and to suggest reporting aspects that should be addressed in the supporting E&E document. We held a full day meeting on 8 November 2017 in London attended by 27 delegates from the UK, USA, Europe, and Asia. Delegates from the public sector and industry included clinical investigators, trial statisticians, journal editors, systematic reviewers, funding panel members, methodologists, and the CONSORT Executive Group representative. Professor Deborah Ashby was the independent chair of the meeting. We took notes during the meeting and audio-recorded and transcribed the discussions to ensure that the content was accurately captured. Following the discussion of each checklist item or group of checklist items, we asked delegates to anonymously vote about the inclusion of a specific item; ‘keep’, ‘drop’, and ‘unsure or no opinion’. We also included the item-voting preferences of a 28th delegate who was unable to attend in person but provided their ratings of checklist items remotely and the project support administrator voted on their behalf. Twenty-six delegates voted, with EC and the independent chair excluded from voting to maintain the independence of the process.

## Results

### Response rates across rounds

In round 1, we registered 143 participants, 94 (65.7%) completed the survey. Of these 94, 86 (91.5%) rated all 58 items and the remaining 8 (8.5%) rated 45 items or fewer. We registered an additional 13 participants after round 1, bringing the total registered participants in round 2 to 156. The round 2 response rate was 114/156 (73.1%). Of these 114, 110 (96.5%) rated all 38 items and the remaining 4 (3.5%) rated 22 items or fewer.

Excluding 13 participants who were only registered after round 1, 79/143 (55.2%) completed both round 1 and 2 surveys. Of the 114 round 2 responders, 35 (30.7%) did not complete the round 1 survey.

### Characteristics of registered participants and responders

Additional file 5 presents the demographics and characteristics of registered participants and responders (completers of at least one reporting item in at least one round). Registered participants and responders were very similar across rounds. Responders in rounds 1 and 2 were based in 19 and 21 countries, respectively; the majority were from the UK, other European countries, and the USA. The majority of responders identified themselves as statisticians in their primary role in trials research; other prominent roles were clinical investigators and trial methodologists. However, the secondary roles in trials research were more diverse. Some stakeholder groups including regulatory assessors, health economists, and research ethicists were underrepresented. Over two thirds of responders were from the public sector. Responders had diverse AD-related experience, and most identified themselves as clinical trialists or methodologists.

### Delphi round 1

#### Perceptions of proposed items

Additional file 6 summarises the distribution of the responders’ perceptions of the importance of reporting items. Detailed item descriptors are accessible online (see download at 10.15131/shef.data.6198290). Of the 22 new items, 11 (50.0%) and 17 (77.3%) were perceived as critical for inclusion by at least 70% and 50% of responders, respectively. Except for one modified item (15a—appropriate baseline data for comparability), which was rated as critical by only 62.9% of responders, the remaining 14 modified items were rated as critical by at least 70% of responders.

**Fig. 4:**
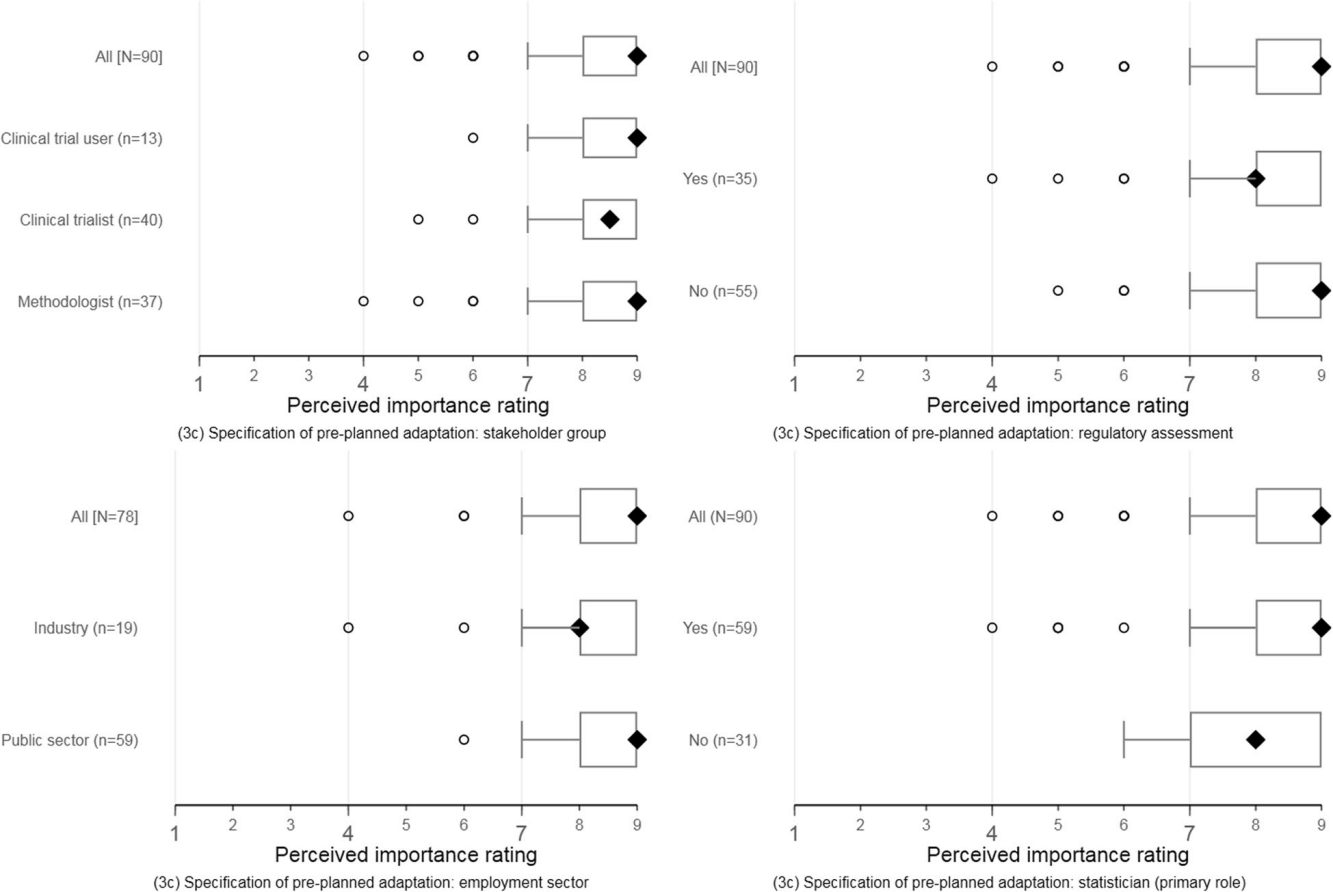
Round 1 perceptions about the importance of specifying pre-planned adaptations (item 3c). Item descriptor is downloadable at 10.15131/shef.data.6198290

**Fig. 5:**
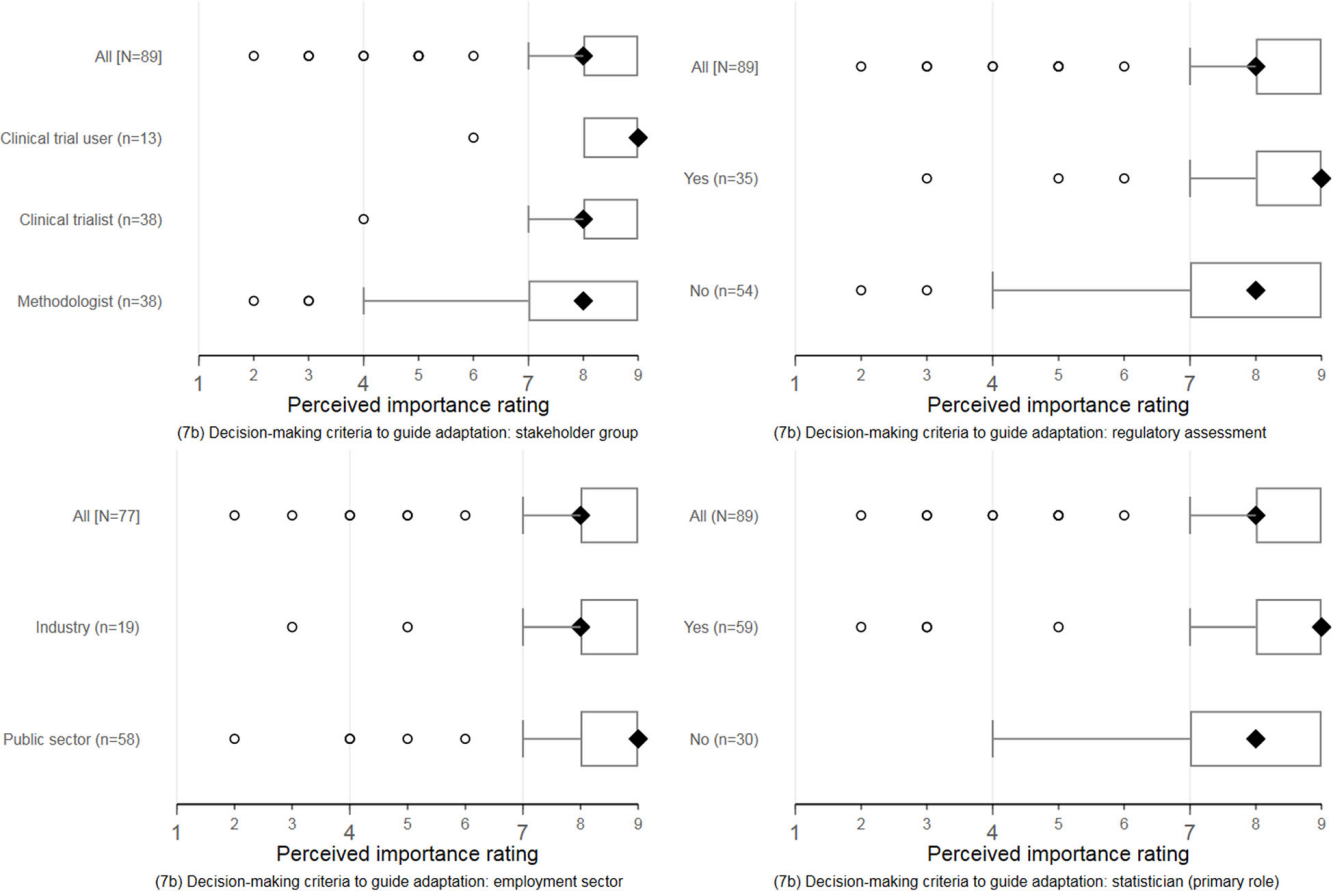
Round 1 perceptions about the importance of decision-making criteria to guide adaptation (item 7b). Item descriptor is downloadable at 10.15131/shef.data.6198290

The perceptions of responders about the importance of suggested reporting items were broadly consistent across self-identified stakeholder groups, employment sectors, regulatory assessment experience, and statistical primary role. Figures 4 and 5 display these response patterns for two reporting items selected for illustration. The remaining clustered boxplots for the new or modified items are accessible online (see download at 10.15131/shef.data.6139721.v1).

#### Open-ended feedback from participants and Steering Committee decisions

On 3 July 2017, the SC met face-to-face to discuss the round 1 Delphi survey results. The summary of the open-ended feedback we received is accessible online (see download at 10.15131/shef.data.6139631). Some responders highlighted that the guideline does not cover ADs used in non-randomised studies. However, we intentionally restricted the scope of the guideline to randomised trials to conform to the scope of CONSORT 2010 framework and to avoid additional complexities. We suggest a separate reporting guideline specific to non-randomised ADs commonly applied in phase 1 trials.

In the feedback, some responders were concerned that the draft checklist included little about aspects relating to Bayesian AD trials. The SC had thought about this at the planning stage and decided to make this guideline as general as possible and applicable to all AD randomised trials regardless of whether they were designed and analysed using frequentist, Bayesian, or both statistical paradigms. The E&E document will further discuss the scope of the guidance and illustrate reporting using examples of various frequentist and Bayesian randomised trials that use an AD.

In general, the qualitative feedback acknowledged that the first checklist draft was comprehensive. However, some responders felt that there were too many items which may impede the use of ADs. The feasibility of reporting all aspects due to limited journal space was questioned although this should no longer be a barrier to complete reporting due to the availability of online repositories. However, the SC deliberately included a large number of draft items at this stage of the Delphi survey to gather perceptions about their importance. The aim of the Delphi process and the subsequent consensus meeting was then to help the SC to decide on essential items to retain.

Some responders suggested the need to include aspects of an estimand of interest, such as under item 2b (specific objectives and hypotheses). The SC acknowledge that the importance of estimands is growing [71–73]. It was felt that estimands are applicable to every trial, and therefore, we recommended via the CONSORT Executive Group representative that such a modification should be considered as a general amendment to the standard CONSORT 2010 when it is revised.

Based on the findings and feedback gathered, the SC made the following key decisions:

- Open registration of new participants prior to round 2 specifically targeting journal editors to improve their participation;
- Exclude the rating of unchanged items in round 2 to shorten completion time but include these items in the survey only to gather any qualitative feedback;
- Terminate the Delphi survey after round 2 because the ratings suggested it was unlikely that additional valuable feedback would be gathered after this stage;
- Submit an ethics amendment to increase the number of survey reminders sent out to non-responders to six and the completion period by 1 to 2 weeks in order to improve the response rate;
- Provide general and itemised feedback to responders summarising their feedback and the SC’s response (what you said and what we did/will do);

Additional file 4 summarises some of the SC’s responses to responders’ qualitative feedback.

### Delphi round 2

Additional file 7 presents the summary of item ratings of round 2 survey responders for new and modified items. See download at 10.15131/shef.data.6198347, for the detailed description of items for the main and abstract draft checklists used in round 2.

#### Perceptions of proposed items

For the abstract checklist, 65.8% of responders rated a new item on ‘adaptation decisions made’ as critical for inclusion (Additional file 7). The remaining four modified abstract items were rated as critical by at least 70% of responders. The overall distributions of ratings were relatively similar across these five abstract items.

For the main checklist items (Additional file 7), more than 70% of responders perceived 25/33 (75.2%) of the new or modified items as critical for inclusion, including 18/33 (54.5%) that were rated as critical by more than 90% of responders. Only 4/33 (12.1%) items received less than 50% votes for being critical: contribution to future research (22b), simulation protocol and report (24d), data monitoring committee charter (24e), and statistical code (24f). However, these items were perceived as at least important by more than 80% of responders. The remaining four items were perceived as critical by between 60% and 68% of responders: dealing with overrun trial participants (12e), representativeness of patient population (15b), access to intentionally withheld information during trial conduct (24b), and access to the statistical analysis plan (24c).

As in round 1, the perceptions of responders about the importance of suggested reporting items were broadly consistent across self-identified stakeholder groups, employment sectors, regulatory assessment experience, and statistical primary role. Clustered boxplots showing response patterns in item ratings are accessible online (see download 10.15131/shef.data.6139721.v1).

For each item, we calculated the proportion of responders who did not change their item ratings between rounds. The median (IQR) of these item rating proportions was 54.1% (48.6% to 57.1%) with a range of 38.7% to 61.6%. Individual item ratings between rounds were broadly consistent (Additional files 8 and 9). In addition, on average, most responders who changed their rating in round 2 increased scores from round 1 except for items 22b (contribution to future research) and 24e (data monitoring committee charter) (Additional file 9).

#### Open-ended feedback from participants

A summary of the open-ended feedback received in round 2 of the Delphi survey that was reviewed during the consensus meeting is accessible online (see download at 10.15131/shef.data.6139631). Two responders queried whether it was important to identify a trial as ‘adaptive’ in the title. We agreed on the importance of indexing an AD trial as adaptive. However, due to the increasing number of guidelines, it is impractical to mandate keywords in the title for every trial publication. Instead, we decided to recommend the inclusion of the word ‘adaptive’ in the trial abstract or at least as a keyword. This simplifies the search for AD trials in literature databases. A new item 3c (specification of pre-planned adaptation) then captures the details about the AD used.

### Consensus meeting discussions

For the main checklist, Table 1 summarises the ACE Consensus Group discussions and advisory decisions made with suggestions of related issues to address. Delegates voted whether to keep or discard each item or whether they were unsure. There was a consensus (≥ 70% of votes) to include ten AD-specific items in the main checklist guideline, of which five were new and five modified items. A further five items were favoured by at least 50% of delegates: AD properties (50.0%), sample size (65.6%), and 53.8% each for randomisation updates after trial commencement (8c), dates defining periods of recruitment (14a), and for the inclusion of the statistical analysis plan (24c). A suggestion was made to expand the explanatory text of the E&E document for six items to clarify additional requirements for some ADs without modifying the item: items 14b (unexpected termination/why the trial ended or was stopped), 15 (appropriate baseline data for comparability), 16 (numbers analysed at interim and final analysis), 17a (primary outcome results), and 20 (limitations, sources of bias, imprecision and deviations) and 21 (generalisability) (Table 1). It was apparent after the meeting that modified item 6b (unplanned changes to outcomes) and new item 14c (adaptation decisions) that were supported by 46.2% of votes for inclusion needed further discussions by the SC (Table 1).

**Table 1:** Consensus meeting discussions and advisory decisions for the main checklist reporting items

Main checklist item	Summary of the discussion and advisory decisions and suggestions made
3a Description of the trial design	Queried the need for the modification. However, it was noted that the allocation ratio for some ADs can change over time and therefore needs greater prominence or some alternative language in the E&E document to indicate that the initial allocation ratio can be fixed or change during the course of the trial Decision: *16 (61.5%) voted to drop the modification and keep the original item*
3b Rationale for an AD	Noted the importance of the rationale especially when interacting with key stakeholders particularly at the planning stages. In addition, from a regulatory perspective, a well-explained rationale is important in the evaluation process. However, the need for a standalone item covering the rationale for the AD was questioned, especially given that no justification for fixed sample size designs is required. Some suggested to drop 3b as a standalone item but make it clear in the E&E document that 2a ‘scientific background and explanation of the rationale’ should also be about ‘scientific rationale for choosing an AD’ Decision: *17 (65.4%) voted to drop the standalone item and find a compromise solution*
3c Specification of pre-planned adaptations	Described as the essential part of the trial design. The importance of separating planned adaptations from unplanned changes was highlighted. It was suggested that the new text from 3a could be added to 3c to ensure that all material is adequately covered Decision: *21 (80.8%) voted to keep the new item as standalone*
3d Unplanned changes to the trial design or methods	Importance of covering both planned adaptations and unplanned changes adequately, as this is key to ADs. Decision: *21 (80.8%) voted to keep the modified item as standalone and address ordering issues*
3e Adaptive design properties	Importance of AD properties from a regulatory perspective was highlighted. Noted that statistical and operational properties of the ADs are broad and include sample size. There was a discussion about whether the AD properties should be covered here or under sample size (7a) since the aspects covered by 3e and 7a overlap but not identical. Some suggested this should be under the section heading ‘Sample size and operating characteristics’. Some felt that 3e, 7a, and 7c overlap, so some further work is needed to clarify this. Decision: *13 (50.0%) voted to keep the item and address structural issues*
6a Pre-specified outcomes	Discussion reflected that a trial could be adapted based on an ‘intermediate’ outcome that may or may not be a secondary outcome. The need for ‘clinical rationale’ was questioned and clarification given as it relates to the rationale for selecting an ‘intermediate’ outcome to adapt the trial or help make interim decisions. Some suggested rewording to ensure that pre-specified primary and secondary outcomes, together with additional ‘intermediate’ outcomes are all adequately covered. The complexity of material covered by this item was acknowledged. Decision: *23 (88.5%) voted to keep the modified item and address wording issues*
6b Unplanned changes to outcomes	Mixed views on the necessity of the modification. Some were concerned that this part of studies is often poorly reported. Some concern that modifying the item may obscure the original meaning. Reflecting on the discussion, we failed to clarify that some AD trials can change the outcome in a planned manner reflected under 3c. To retain the intention of the original item, this modification a clarification to capture unintended changes to outcomes (outside the scope of the planned adaptations) Decision: *12 (46.2%) voted to keep the modified item. Steering Committee to review*
7a Sample size	Mixed views on whether the modification was necessary or proportionate. Suggestions that the wording could be simplified or shortened so that content on sample size is not obscured. For example, by adding AD properties, as per discussion of 3e above, modifying section heading, and further details would be better added to the E&E document Decision: *17 (65.4%) voted to keep the modified item and address raised issues*
7b Decision-making criteria to guide trial adaptation	Importance acknowledged and suggestions to simplify the wording and discuss details in the E&E document Decision: *23 (88.5%) voted to keep the modified item and address wording issues*
8c Randomisation updates after trial commencement	Importance reflected in the discussion and suggestions to merge the material of items 8b and 8c Decision: *16 (61.5%) voted to discard this item as a standalone, but keep the content of the suggested new item by merging with item 8b*
11c Confidentiality and minimisation of operational bias	General agreement that the information included was essential Decision: *26 (100.0%) voted to keep the new item as a standalone*
12c Methods for statistical inference; 12d Methods to combine data across stages; 12e Dealing with over-run participants; 12f Methods for dealing with multiple treatment comparisons/outcomes; 12g Prior selection	Extended discussions about whether the material covered by 12c–12g should be addressed via individual checklist items or a merger. Some concern that the use of a long, compound item would not improve the quality of reporting, as authors retreat to the bare minimum to complete the checklist. Cross-referencing the protocol or the statistical analysis plan was suggested to capture the complexity of statistical inferential methods in the AD. Some suggested discussing the potential sub-items in the E&E document. Discussed whether the approach to methods used for futility analyses should be covered here; some suggested rewording 3c such that it also captures methods used to derive statistical information used to adapt a trial. Acknowledged the need to reword the material such that key aspects of the dropped items 12d–12g are reflected in some way Decisions: *25 (96.2%) consensus to keep 12c but address rewording and reflection of dropped items* *21 (80.0%) consensus to drop 12d as a standalone item* *22 (84.6%) consensus to drop 12e as a standalone item* *20 (76.9%) consensus to drop 12f as a standalone item* *21 (80.8%) consensus to drop 12g as a standalone item*
13a Randomised, received intended treatment…	Extended discussions about the definition of ‘intermediate’ and interim outcomes/analysis and need for simplification. Discussion on the meaning of ‘subpopulations’ and its limited applicability to population enrichment designs, which suggests it should be removed and discussed in the E&E document. Mixed views expressed on coverage of reporting and whether this can be differentiated for all adaptations; graphical complexities highlighted for some trial adaptations Decision: *20 (76.9%) consensus to keep the modified item and address rewording*
14a Dates defining recruitment periods	Important to ensure the meaning of the original 14a is not lost with respect to study dates Decision: *14 (53.8%) voted to keep the modified item*
14b Unexpected termination	Decision: *17 (65.4%) voted to drop the modification and keep the original item*
14c Adaptation decisions	Acknowledged that adaptation dates and decisions should be included, but query on whether items 14a and 14c are the correct place and need for rewording. Discussion on the need for implementation resources to help with reporting. Some confusion about details/coverage of item 14c evident in discussions Decision: *12 (46.2%) voted to keep the item. Steering Committee to review*
15a Appropriate baseline data for comparability	The necessity of modifying 15a was queried and the need to reword ‘subpopulation’. Query over whether using baseline is correct, but acknowledged that changing this would require a change to CONSORT 2010. Suggestion to drop extended 15a but include an explanation of the need to present information differently for some AD trials such as population enrichment, with an example in the E&E document Decision: *13 (50.0%) voted to drop an item and consider expanding the explanatory text*. *Steering Committee to review*
15b Representativeness of patient population	No specific issues raised *Decision: 20 (76.9%) consensus to keep the new item*
16 Numbers analysed at interim and final analysis	The distinction for AD trials with examples could be adequately covered in the E&E document without the need to modify the original item wording Decision: *19 (73.1%) voted to drop the item and expand the explanatory text*
17a Primary outcome results 17c Suitable representation of interim outcome results	Suggestion that it is unnecessary to modify 17a, but rather ensure that new material is all captured under 17c. Queries about whether CONSORT 2010 already covers the relevant content, and whether additional content is just required for the E&E document (similar to items 15a and 16). Highlighted the importance of understanding important changes relative to the feasibility of covering all adaptation aspects within a standard journal article. Importance of transparency about the location of more detailed analyses was suggested as a compromise—it is more about access to key information. Suggestion that reporting of treatment arms that have been dropped should be mandatory. Decisions: *16 (61.5%) voted to drop item 17a and expand the E&E text* *21 (80.8%) consensus to retain 17c but needs rewording*
20 Limitations, sources of bias, imprecision, and deviations	Questioned the necessity of the modification given that the original item is already broad. Making reporting more stringent for ADs relative to fixed sample size designs was questioned. Clarification of AD-related issues could be given in the E&E document without the need to reword the item Decision: *25 (96.2%) voted to drop the item and expand the explanatory text*
21 Generalisability (external validity and applicability)	The necessity of modification queried on the ground that this is too specific to only a small type of ADs such as population enrichment. Again, clarification of AD-related issues could be given in the E&E document without the need to reword the item Decision: *25 (96.2%) voted to drop the item and expand the explanatory text*
22b Contribution to future-related research	Consensus group appreciated the sentiment of this new item, but the necessity was strongly questioned Decision: *23 (88.5%) voted to drop the item*
24b Intentionally withheld information 24c Statistical analysis plan 24d Simulation protocol and report 24e Data Monitoring Committee Charter 24f Statistical code	Discussed the feasibility and necessity of including all proposed individual items, and whether these could be merged into one larger item covering additional trial information. Some delegates felt that 24b and 24c should be kept as standalone items and merge 24d to 24f under the heading ‘Availability of other trial documents, if available’ Decisions: *17 (65.3%) voted to keep item 24b as a standalone and merge with 24d, 24e, and 24f* *14 (53.8%) voted to keep item 24c as a standalone and address merging issues*

For the abstract (Table 2), there was an agreement to include two modified items (description of trial design and clearly defined outcome for this report) and one new item (adaptive decisions made). A recurrent discussion point was the need to minimise adding new items to the abstract unless they are essential due to word limits imposed by journals.

**Table 2:** Consensus meeting discussions and advisory decision for the abstract checklist reporting items

Abstract item	Summary of the discussion and advisory decisions and suggestions
1b Description of trial design	Query about feasibility including detailed AD features in limited word count for abstracts. Debated the use of term ‘adaptive’ in the abstract to help identify these trials; care should be taken as there is a grey area around the classification of some group sequential designs as ADs in some quotas. Some suggested making a distinction between trials where the only adaptation is to stop the whole study and other ADs that must use the term adaptive in the abstract. The E&E could address the scope by highlighting the type of ADs. Decision: *21 (80.8%) consensus to keep the modified item but reword*
1c Clearly defined outcome for this report	Importance of describing adaptive outcome used to aid credibility of results and help with locating AD trials. Replace the term ‘intermediate’ outcomes consistent with earlier discussions Decision: *19 (73.1%) consensus to keep the modified item but reword*
1d Result for each group	Concern expressed about the feasibility of including results for each outcome in the abstract. Discussion around the necessity of including results for primary and intermediate results, particularly where the latter are used as the basis for adaptation decisions. Concerns about confusion in terminology (‘interim’ and ‘intermediate’) Decision: *21 (80.8%) consensus to drop the modified item and keep the original*
1e Adaptive decisions made	Several participants acknowledged the importance of this item but queried coverage of reporting. Helpful for literature searching to identify specific trials. Important to ensure that authors indicate where no changes or adaptations made. Suggestion to cover the checklist earlier before outcomes. Noted results inconsistencies between items 1e and 14c although it is the same item—perhaps due to the confusion highlighted under item 14c Decision: *23 (88.5%) consensus to keep the new item*
1f Conclusions	For consistency with earlier items (21 and 22), the group acknowledged that this item should not be extended Decision: *22 (84.6%) consensus to drop the modified item and keep the original*

### Finalisation of the checklist

On 1 February 2018, the SMG met to discuss advisory decisions and suggestions made at the consensus meeting. The group discussed each item reflecting on the consensus report and agreed on the items to retain and structural changes required in the guidance.

The advisory decisions and suggestions from the consensus meeting were taken on board. The rationale for an AD (item 3b, Table 1) was dropped as a compromise but will be discussed in the E&E text under item 3c (pre-planned adaptations) and linked to the scientific background and explanation of the rationale (item 2a). We merged items 3e (AD properties) and 7b (sample size) because they are connected. As a result, we renamed the ‘sample size’ subheading to ‘sample size and operating characteristics’. The modified item 6b (unplanned changes to outcomes) with borderline results was included for clarification purpose. In addition, item 14c (adaptation decisions) was discussed as very important and also included for consistency with the abstract decisions. For items 24b to 24f (Table 1), we decided to keep the statistical analysis plan (24c) as an important standalone item and merge to include other items (24b intentionally withheld information, 24d simulation protocol and report, 24e data monitoring committee charter and 24f statistical code) for discussion in the E&E document for good practice.

For the abstract, we acknowledged the importance of including a clearly defined outcome used to inform adaptation if different from the primary outcome (1c Table 2). However, for parsimony reasons due to word limit imposed on abstracts, we dropped the modified item but will instead expand the E&E text discussing circumstances when this information is desirable to be included in the abstract.

Following the meeting, the checklist was revised including rewording and reordering of some items (such as item 3c ‘specification of pre-planned adaptation’ to 3b ‘pre-planned adaptive design features’) in consultation with the SC. On 13 March 2018, we shared the revised checklist with the ACE Consensus Group for their final feedback on the changes made. On 18 April 2018, we finalised the ACE main and abstract checklists that were signed off by the ACE Consensus Group which will be presented in the forthcoming E&E document. The ACE main checklist contains seven new and nine modified items, as well as six unchanged items that were recommended for inclusion in the expanded text of the E&E document for clarification. The other 21 items remain unchanged from the CONSORT 2010 Statement. The ACE abstract checklist had one new item, one modified item, and an unchanged item with expanded text, as well as 15 unchanged items. Table 3 presents the finalised modifications to the abstract and main report checklists excluding unchanged items.

**Table 3:** Finalised CONSORT extension for adaptive design randomised trials (only new and modified items and those with expanded E&E text)

Section/topic by item no	Standard CONSORT for abstracts and conference posters [84, 85]	Abstract extension for adaptive design randomised trials
Title and abstract		
Trial design	Description of the trial design (for example, parallel, cluster, non-inferiority)	Description of the trial design (for example, parallel, cluster, non-inferiority); include the word ‘adaptive’ in the content or at least as a keyword
Outcome	Clearly defined primary outcome for this report	[expand E&E text for clarification]
Adaptation decisions made		Specify what trial adaptation decisions were made in light of the pre-planned decision-making criteria and observed accrued data
Section/topic by item no	Standard CONSORT 2010 checklist item [37, 65]	Main report extension for adaptive design randomised trials
Trial design		
3b « pre-planned adaptive design features		Type of adaptive design used, with details of the pre-planned trial adaptations and the statistical information informing the adaptations
3c « 3b	Important changes to methods after trial commencement (such as eligibility criteria), with reasons	Important changes to the design or methods after trial commencement (such as eligibility criteria) outside the scope of the pre-planned adaptive design features, with reasons
Outcomes		
6a	Completely defined pre-specified primary and secondary outcome measures, including how and when they were assessed	Completely define pre-specified primary and secondary outcome measures, including how and when they were assessed. Any other outcome measures used to inform pre-planned adaptations should be described with the rationale
6b	Any changes to trial outcomes after the trial commenced, with reasons	Any unplanned changes to trial outcomes after the trial commenced, with reasons
Sample size and operating characteristics		
7a	How sample size was determined	How sample size and operating characteristics were determined
7b	When applicable, explanation of any interim analyses and stopping guidelines	Pre-planned interim decision-making criteria to guide the trial adaptation process; whether decision-making criteria were binding or nonbinding; pre-planned and actual timing and frequency of interim data looks to inform trial adaptations
Sequence generation		
8b	Type of randomisation; details of any restriction (such as blocking and block size)	Type of randomisation; details of any restriction (such as blocking and block size); any changes to the allocation rule after trial adaptation decisions; any pre-planned allocation rule or algorithm to update randomisation with timing and frequency of updates
Blinding		
11c Confidentiality and minimisation of operational bias		Measures to safeguard the confidentiality of interim information and minimise potential operational bias during the trial
Statistical methods		
12a	Statistical methods used to compare groups for primary and secondary outcomes	Statistical methods used to compare groups for primary and secondary outcomes, and any other outcomes used to make pre-planned adaptations
12b « Estimation and inference methods		For the implemented adaptive design features, statistical methods used to estimate treatment effects for key endpoints and to make inferences
Participant flow (a diagram is strongly recommended)		
13a	For each group, the numbers of participants who were randomly assigned, received intended treatment, and were analysed for the primary outcome	For each group, the numbers of participants who were randomly assigned, received intended treatment, and were analysed for the primary outcome and any other outcomes used to inform pre-planned adaptations, if applicable
Recruitment and adaptations		
14a	Dates defining the periods of recruitment and follow-up	Dates defining the periods of recruitment and follow-up, for each group
14b	Why the trial ended or was stopped	[expand E&E text for clarification]
14c Adaptation decisions		Specify what trial adaptation decisions were made in light of the pre-planned decision-making criteria and observed accrued data
Baseline data		
15a « 15	A table showing baseline demographic and clinical characteristics for each group	[expand E&E text for clarification]
15b Similarity between stages		Summary of data to enable the assessment of similarity in the trial population between interim stages
Numbers analysed		
16	For each group, number of participants (denominator) included in each analysis and whether the analysis was by original assigned groups	[expand E&E text for clarification]
Outcomes and estimation		
17a	For each primary and secondary outcome, results for each group, and the estimated effect size and its precision (such as 95% confidence interval)	[expand E&E text for clarification]
17c Interim results		Report interim results used to inform interim decision-making
20 Limitations	Trial limitations, addressing sources of potential bias, imprecision, and, if relevant, multiplicity of analyses	[expand E&E text for clarification]
21 Generalisability	Generalisability (external validity, applicability) of the trial findings	[expand E&E text for clarification]
Statistical analysis plan and other relevant trial documents		
24b		Where the full statistical analysis plan and other relevant trial documents can be accessed

‘X « Y’ means original item Y has been renumbered to X; ‘X «’ means reordering resulted in the new item X replacing the number of the original item X; [expand E&E text for clarification] means we retain the original item but will discuss additional considerations relating to specific adaptive designs for clarification in the forthcoming explanation and elaboration (E&E) document

## Discussion

### Main results or outputs

We have developed a consensus-driven extension to the CONSORT 2010 Statement for randomised trials using an AD to enhance transparency and adequate reporting. In the spirit of transparency, we have described in this paper the process for the development of the ACE checklist and provided all supporting information that aided the decision-making process. We hope that our experiences can help others in the development of other guidelines or extensions.

The guideline aims to promote transparency and adequate reporting of randomised trials that use ADs and not to stifle design innovation or application of ADs. The ACE checklist provides the minimum requirements that we encourage researchers to report. It is good scientific practice to present additional information beyond this guideline if it helps the interpretation of AD trial results. In principle, we are not advocating the inclusion of details of every trial aspect in a single journal publication. We believe that the most important is the access to details relating to the identified reporting items. For example, researchers can cite other accessible sources of information such as the protocol, simulation protocol and report, a prior publication detailing study design and rationale, methodology publications, and supplementary materials. In addition, the publishing landscape is rapidly changing to meet the needs for more transparency and adequate reporting.

During the development process, the SC came across a few reporting aspects that could be changed or added such as on estimands [71, 72] and data transparency but decided not to do so. This is because we felt that changes to reporting aspects that apply to every trial should be managed via universal amendments to the CONSORT 2010 Statement. We did not want the ACE to selectively put additional hurdles on ADs on reporting aspects which would also apply for other fixed designs. We have communicated this decision to the CONSORT Executive Group through its SC representative.

The ACE reporting guideline is applicable to all randomised AD trials regardless of the statistical framework used to design and analyse the trials (frequentist, Bayesian, or both). The supporting E&E document to be accessed via the CONSORT [74] and EQUATOR Network [42] websites will explain the checklist items in detail with the aid of examples and discussion. The E&E document will guide study publication authors in determining which minimum AD aspects warrant reporting and in what level of detail under different circumstances aided by examples. We hope this ACE reporting guideline will address some concerns about certain AD trials and, consequently, indirectly improve their design, conduct, and interpretability of results. We encourage researchers to use the guideline and journal editors and reviewers to enforce compliance as part of their publication policy. The usefulness of reporting guidelines can be maximised when there are adequate processes in place to enforce their compliance [75].

### Main strengths

We used a consensus-driven Delphi methodology recommended when developing healthcare reporting guidelines [39]. We engaged with key stakeholders in trials research and potential end-users of the resultant ACE reporting guideline throughout the development process that involved participants from a wide range of scientific disciplines, employment sectors, and nationalities with diverse AD-related experiences. Throughout the checklist drafting process, an External Expert Panel provided quality control assurances. Given the topic of the guideline, we adapted the development process in response to the feedback gathered. The CONSORT Executive Group through its representative (DA) oversaw the development process of the guideline throughout. This research developed a CONSORT extension for AD randomised trials using this robust approach endorsed by the CONSORT Executive Group.

We recorded high response rates of 94 (66%), 114 (73%), and 79 (55%) in round 1, round 2, and across both rounds of the Delphi survey, respectively. The number of registered participants and responders is larger than other similar Delphi surveys [62, 76, 77]. The characteristics and demographics of registered participants and responders were very similar across Delphi survey rounds. In addition, the number of registered participants and responders is larger than in most Delphi surveys used to develop healthcare reporting guidelines [78, 79] and comparable to the one of the latest guideline on pilot and feasibility studies [80, 81]. We also improved the participation of key end-users of the guideline (journal editors) in round 2 by reopening registration after round 1. Finally, we achieved a high degree of consensus that was consistent across Delphi survey rounds for the majority of the items. Additional supplementary materials are publicly accessible (Additional file 10) including participants who took part (Additional file 11).

### Main limitations

Despite the highlighted strengths of this study, we also identified a number of limitations. First, over half of the survey participants were statisticians in their primary role in trials research and even though industry currently contributes a huge proportion of ADs [18–20, 28, 82], over two thirds of participants were employed in the public sector. However, the secondary roles of participants in trials research were more diverse including clinical investigators and trial methodologists. Nonetheless, perceptions about the importance of items were broadly consistent regardless of the primary roles of the participants, and their self-identified stakeholder group, and employment sector.

Second, despite our broad engagement efforts, the number of participants from some stakeholder groups was small such as health economists, regulatory assessors, and research ethicists. Research on obstacles to AD trials also reported poor uptake among these stakeholder groups [26, 28]. The implications for the guideline development are unclear. Paradoxically, although few participants identified themselves as regulatory assessors, about 43% stated that they had AD-related regulatory assessment experiences. This could include researchers with regulatory experiences as part of regulatory engagements or submissions of their trials, previous employees of regulatory agencies, or current regulatory assessors who did not want to identify themselves as employees of regulatory agencies during the surveys due to contractual issues. However, the perceptions of responders were consistent regardless of the stated AD regulatory assessment experiences. It should also be noted that there was only a small number of regulatory assessors available for the sampling frame.

Finally, for practical purposes in line with the CONSORT 2010 statement, the ACE reporting guideline applies to randomised trials that use ADs. Hence, the guideline does not specifically address reporting aspects of non-randomised AD studies that are also applied in early phase trials. Nevertheless, the basic principles of the ACE reporting guideline may still be applicable to these interventional studies and are consistent with some researcher good practice propositions for writing early-phase AD study protocols [83]. We believe there is scope for a consensus-driven approach to develop a reporting guideline for non-randomised AD studies.

## Conclusions

We have developed a consensus-driven CONSORT extension for AD randomised trials. This paper transparently describes how we reached the final ACE reporting checklist and the forthcoming E&E document and provides all supporting information that aided the decision-making process. The process we described is not just applicable to ADs, and so we hope this will help researchers in the development of future guidelines or extensions to learn from our experiences. The ACE reporting guideline is applicable to all AD randomised trials and contains minimum reporting requirements with appropriate flexibility to be described in the E&E document. We hope the guideline will improve the reporting of AD randomised trials, enhance their interpretability and credibility of their results, improve their reproducibility, and indirectly facilitate their robust design and conduct.

## Box 1 ACE project long-term goals

▪ To enhance transparency and adequate reporting of randomised trials that use an AD

▪ To improve the usefulness of randomised trial case studies that use an AD and bridge the gap in practical knowledge

▪ To mitigate some concerns about AD methods and enhance the interpretability and the credibility of results from randomised trials that use an AD

▪ To indirectly enhance robust design and conduct of randomised trials that use an AD

▪ To enhance the reproducibility of randomised trials that use an AD and reduce research waste

## Box 2 Themes that may influence reporting of ADs

1. The risk of introducing operational bias into the conduct of the trial increases when interim data are reviewed.

2. Performing multiple hypotheses tests increases the risk of making inappropriate or unjustified claims about the treatment effect if inappropriate statistical methods are used. This occurs for example when conducting interim analyses; evaluating multiple patient subgroups, treatments, or endpoints; or a combination of these.

3. Not addressing planned adaptations and unplanned changes may potentially invalidate results depending on their form, purpose, and the statistical methods used.

4. Biased estimates of the treatment effect may be produced if inappropriate statistical methods are used for analysis.

5. The risk of making undesirable trial adaptation decisions based on premature or inadequate interim data.

6. The type of adaptive design, form of trial adaptations, and interim adaptation decision rules used may influence the acceptability of results, level of information to disclose, and the applicable statistical methods.

7. Unintended changes in patient characteristics or the estimand (what is to be estimated) before and after trial adaptation may occur, making overall results difficult to interpret.

8. The need for more transparency to enable research consumers to evaluate the appropriateness of the methods, sources of potential bias, and interpretability and trustworthiness of the trial results, as well as to enable other researchers to reproduce trial-related aspects.

## Additional files

Additional file 1: ACE project management activities. Summary of ACE project management related activities during the development process. (DOCX 21 kb)

Additional file 2: Definitions of technical terms relating to adaptive designs. List of adaptive designs technical terms and definitions agreed by the ACE Steering Committee. (DOCX 26 kb)

Additional file 3: Platforms used to reach out to key stakeholders for Delphi surveys. List of platforms used to reach out to key stakeholders for Delphi surveys. (DOCX 20 kb)

Additional file 4: Qualitative feedback from round 1 Delphi survey and our response. Qualitative feedback and Steering Committee responses. (DOCX 20 kb)

Additional file 5: Demographic and characteristics of registered participants and responders. Summaries of Delphi survey participants. (DOCX 23 kb)

Additional file 6: Round 1 summary of perceptions about the importance of reporting items. Summaries of round 1 perceptions about the importance of reporting items. (DOCX 28 kb)

Additional file 7: Round 2 summary of perceptions about the importance of reporting items. Summaries of round 2 perceptions about the importance of reporting items. (DOCX 29 kb)

Additional file 8: Measures of agreement in rating scores between Delphi rounds 1 and 2 survey. Summaries of measures of agreement in the rating of participants between rounds 1 and 2 of the Delphi surveys. (DOCX 24 kb)

Additional file 9: Distributions of the change in rating scores from round 1 and Bland-Altman plots. Bland-Altman plots and histograms showing the distribution of change in scores from round 1. (DOCX 1205 kb)

Additional file 10: Accessible supplementary material hosted within the University of Sheffield ORDA repository. Summary reports; draft checklists used in round 1 and 2 Delphi surveys; registration and Delphi survey rounds datasets; Figures (clustered boxplots) displaying responders’ perceptions of reporting items stratified by key characteristics. (DOCX 22 kb)

Additional file 11: Registered participants for the Delphi surveys. List of participants who registered to take part in the Delphi surveys. This includes only those who did not opt out to be publicly acknowledged. (DOCX 19 kb)

## Abbreviations

ACE: Adaptive designs CONSORT Extension
AD: Adaptive design
ADMTP: Adaptive Designs and Multiple Testing Procedures
ADWG: Adaptive Designs Working Group
CONSORT: Consolidated Standards of Reporting Trials
CTU: Clinical Trials Unit
DIA: Drive Insights to Action
DRF: Doctoral Research Fellowship
E&E: Explanation and elaboration
HTMR: Hubs for Trials Methodology Research
IQR: Interquartile range
ISCB: International Society for Clinical Biostatistics
MRC: Medical Research Council
NIHR: National Institute for Health Research
PhRMA: Pharmaceutical Research and Manufacturers of America
PSI: Statisticians in the Pharmaceutical Industry
REC: Research Ethics Committee
SC: Steering Committee
ScHARR: School of Health and Related Research
SCT: Society for Clinical Trials
SD: Standard deviation
SMG: Study Management Group
UKCRC: United Kingdom Clinical Research Collaboration
