# Supplementary material for: Development process of a consensus-driven CONSORT extension for randomised trials using an adaptive design
Source: BMC Med. 2018 Nov 16;16:210. doi: 10.1186/s12916-018-1196-2 (PMC6238302; doi:10.1186/s12916-018-1196-2)
Supplement: Supplementary file 1 — ACE project management activities. Summary of ACE project management related activities during the development process. (DOCX 21 kb) [file 12916_2018_1196_MOESM1_ESM.docx]

| **Activity** | **Participants** | **Venue and nature of meeting or correspondence** | **Date** |
| --- | --- | --- | --- |
| Overall project planning | Steering Committee | Sheffield  (face-to-face and teleconference) | 25 October 2016 |
|  |  |  |  |
| Review of the scoping review findings, drafting of adaptive design definition, and preliminary ACE checklist items | Steering Committee | Sheffield  (face-to-face) | 19 January 2017 |
|  |  |  |  |
| Review of the January meeting report and planning of the Delphi surveys | Study Management Group | Sheffield  (face-to-face and teleconference) | 20 February 2017 |
|  |  |  |  |
| Iterative development of the ACE checklist for inclusion in Round 1 of Delphi survey | Study Management Group | Sheffield  (face-to-face and teleconference) | 3 April 2017 |
|  |  |  |  |
|  | External Expert Panel and Steering Committee | Email correspondence | April to May 2017 |
|  |  |  |  |
| Discussion of the Delphi Round 1 survey, progress in the registration of participants, survey launch and related issues | Study Management Group | Teleconference | 26 May 2017 |
|  |  |  |  |
| Review of the Round 1 Delphi survey results, discussion of the feedback received and the checklist changes required and related issues | Steering Committee | Sheffield  (face-to-face and teleconference) | 3 July 2017 |
|  |  |  |  |
| Planning of Round 2 of Delphi survey, review of the iterative development of the checklist for inclusion in Round 2 of Delphi survey | Study Management Group | Sheffield  (face-to-face and teleconference) | 21 August 2017 |
|  |  |  |  |
| Iterative development of the checklist | Steering Committee | Email correspondence | August to September 2017 |
|  |  |  |  |
| Planning of the consensus meeting activities | Study Management | Sheffield  (face-to-face and teleconference) | 12 October 2017 |
|  |  |  |  |
| Review of the Round 2 Delphi survey results and iterative development of the ACE checklist for a consensus meeting | Steering Committee | Email correspondence | October 2017 |
|  |  |  |  |
| Full day consensus meeting | Invited delegates | London  (full day face-to-face) | November 2017 |
| Review of the consensus meeting results and iteration of the ACE checklist | Study Management Group | Sheffield  (face-to-face and teleconference) | 1 February 2018 |
|  |  |  |  |
| Finalisation of the ACE checklist | Steering Committee, ACE Consensus Group | Email correspondence | February to April 2018 |
|  |  |  |  |
