# Supplementary material for: Development process of a consensus-driven CONSORT extension for randomised trials using an adaptive design
Source: BMC Med. 2018 Nov 16;16:210. doi: 10.1186/s12916-018-1196-2 (PMC6238302; doi:10.1186/s12916-018-1196-2)
Supplement: Supplementary file 2 — Definitions of technical terms relating to adaptive designs. List of adaptive designs technical terms and definitions agreed by the ACE Steering Committee. (DOCX 26 kb) [file 12916_2018_1196_MOESM2_ESM.docx]

| **Term** | **Definition** |
| --- | --- |
| Allocation rule | How participants are randomised or allocated to treatment arms in a clinical trial |
| Adaptive design (AD) | A clinical trial design that provides pre-planned opportunities to use accumulating trial data to modify aspects of an ongoing trial while preserving its validity and integrity |
| Validity | Relates to the ability to provide correct statistical inference to establish the effects of interventions under study and produce accurate estimates of the effects (point estimates, confidence intervals and p-values), to give results that are convincing to the broader audience (science community and consumers of research findings) |
| Integrity | Relates to minimisation of operational bias, maintenance of data confidentiality, and ensuring consistency in trial conduct (before and after adaptations) for credibility, interpretability and persuasiveness of the trial results |
| AD features or adaptations | Pre-planned optional changes or modifications considered to be made to the aspects of the trial |
| Adaptive decision-making criteria | Elements of decision-making rules describing *how* and *when* the proposed trial adaptations will be utilised, which could be in the form of a ‘decision tree’ or an algorithm |
| Decision rules | Pre-specified set of actions guiding how decisions about implementing the considered trial adaptations are made during the trial given the interim observed data |
| Decision boundaries | Pre-specified limits or parameters used to determine which trial adaptations should be made during the trial. For instance, stopping boundaries that relate to pre-specified limits regarding decisions to stop the trial or treatment(s) early |
| Binding rules | Decision rules that must be followed or adhered to if the design is to retain its statistical properties or operating characteristics such as control of the false-positive error rate inflation |
| Non-binding rules | Decision rules that may be overruled without affecting key statistical properties or operating characteristics of the design such as inflation of the false-positive error rate |
| Blinded estimation | Parameter estimation without the knowledge or use of the treatment allocation |
| Unblinded estimation or review | Parameter estimation or review of the data with the knowledge or use of the treatment allocation |
| Co-primary objectives | Clinical trials for evaluating efficacy and safety of new interventions include multiple objectives. If a trial is designed to achieve all of the primary objectives, multiple primary objectives become co-primary objectives. Failure to achieve any single objective implies that the trial success cannot be concluded |
| Enrichment design | An AD that prospectively uses the characteristics of potential participants to try to select subset(s) of the trial population that are more likely to benefit from effective study interventions (if they exist) than the intervention effect(s) would be if assessed in the whole (unselected) trial population |
| Group sequential design | A multi-stage AD that allows outcomes from study participants to be analysed in batches or stages (at interims) by treatment comparisons during an ongoing trial (interim analysis). Interim decisions can be made to stop the trial or arm(s) of the trial early if the observed treatment effect has crossed some pre-specified stopping boundaries |
| Statistical properties or  operating characteristics | Relates to the behaviour of the design such as sample size, statistical power, false-positive error rate, bias in the estimation of the treatment effect(s), or probability of each adaptation taking place (such as dropping a treatment arm at any interim) |
| False-positive error rate | Chances of making false claims about the benefits (or safety profile) of the treatments under study. This can be defined depending on the nature of the trial and configuration of the null hypotheses, such as pairwise or familywise type I error rate |
| Intermediate outcome | An outcome that is believed to be informative for the definitive clinical primary outcome, likely to predict the benefits or safety profile of study treatments, and can be observed quicker than the definitive clinical primary endpoint for use during an interim analysis to inform trial adaptations |
| Bias | The general systematic tendency for the treatment effect estimates to deviate from their ‘true values’ or the statistical properties of the trial fail to conform to theoretical properties to address the intended research question(s). This could be statistically or operationally driven as defined below |
| Statistical bias | Bias introduced by the design such as a result of changes to aspects of the trial (adaptations) or multiple analysis of accumulating data from an ongoing trial |
| Operational bias | Occurs when knowledge of key trial-related information influences changes to the conduct of that trial in a manner that bias the conclusions made regarding the benefits and/or safety of study treatments |
| Interim stage | Time point at which interim analysis or data look is performed |
| Interim analysis or interim data look | Statistical analysis based on accumulating data of an ongoing trial (before the final analysis) which may or may not involve treatment group comparisons. Trial adaptations are typically performed at an interim analysis |
| Pre-planned adaptations | Trial adaptations that are enacted at the design stage of the trial or before unblinded review of the trial data |
| Type of an AD | The main category used to classify a trial design by its adaptive design features or adaptations. Examples include response-adaptive randomisation, sample size re-estimation, group sequential, operationally or inferentially seamless, multi-arm multi-stage or population enrichment. Some ADs can fall into more than one main category of trial adaptations, such as group sequential with sample size re-estimation |
| Sample size re-estimation | An AD in which outcome data are reviewed at an interim analysis to re-estimate parameters (such as the variance) used in the sample size calculation. Based on this calculation, a decision is made to modify the pre-determined sample size |
| Simulation | A statistical procedure undertaken using a computer program to evaluate/estimate the statistical properties of the design or/and to quantify bias by generating pseudo outcome data according to the design, under a number of scenarios and repeated a large number of times |
| Seamless design | An AD conducted in multiple stages conducted under a single protocol without stopping recruitment between phases, and in which each phase has its own objectives. For example, a seamless phase 2/3 design addresses exploratory (phase 2) and confirmatory (phase 3) objectives in a single trial |
| Operationally seamless | A seamless design in which a trial is conducted in a sequence of phases and aims to address distinct objectives, however, the data from each phase are kept distinct during the statistical analyses. The statistical analysis of the outcome data at each phase is only based on participants who contributed data for that particular phase |
| Inferentially seamless | A seamless design that makes use of the outcome data from participants who contributed to earlier, related phases. These data are included in the statistical analyses at subsequent phases, hence appropriate statistical methods are required to control for false-positive error rate |
| Response-adaptive randomisation | An AD that uses the outcome data (interim treatment effect results) to change or modify the allocation rule for future study participants in that ongoing trial |
| Multi-arm multi-stage | An AD that allows the simultaneous evaluation of study treatments against shared control arm(s). Adaptations are built in over multiple stages and include options to drop arms for lack of benefit or poor safety profile |
