# Supplementary material for: Development process of a consensus-driven CONSORT extension for randomised trials using an adaptive design
Source: BMC Med. 2018 Nov 16;16:210. doi: 10.1186/s12916-018-1196-2 (PMC6238302; doi:10.1186/s12916-018-1196-2)
Supplement: Supplementary file 3 — Platforms used to reach out to key stakeholders for Delphi surveys. List of platforms used to reach out to key stakeholders for Delphi surveys. (DOCX 20 kb) [file 12916_2018_1196_MOESM3_ESM.docx]

- Social media (such as Twitter and LinkedIn);
- Pre-existing email distribution lists (such as MRC HTMR network, NIHR Statistics Group, UKCRC Registered CTU Network);
- ADs working groups in the UK, other EU countries and the USA (such as MRC HTMR ADWG, Drive Insights to Action (DIA) ADWG, PhRMA ADWG and ADMTP);
- Direct emails to known contacts (such as 44 leading medical journal editors);
- Assisted contacts via the SC members (such as for regulatory agencies and research funders); conference engagements (such as Statistics in the Pharmaceutical Industry, PSI; International Society for Clinical Biostatistics, ISCB; and Society for Clinical Trials, SCT);
- Generic email lists such as INVOLVE ([involve@nihr.ac.uk](mailto:involve@nihr.ac.uk) ).
