# Supplementary material for: Development process of a consensus-driven CONSORT extension for randomised trials using an adaptive design
Source: BMC Med. 2018 Nov 16;16:210. doi: 10.1186/s12916-018-1196-2 (PMC6238302; doi:10.1186/s12916-018-1196-2)
Supplement: Supplementary file 4 — Qualitative feedback from round 1 Delphi survey and our response. Qualitative feedback and Steering Committee responses. (DOCX 20 kb) [file 12916_2018_1196_MOESM4_ESM.docx]

| **Item** | **Issues raised in open-ended feedback** | **Steering Committee response** |
| --- | --- | --- |
| 1b | Not all abstract aspects are equally important so it is helpful to give an option to rate individual items contributing to the abstract. some suggested a number of additions | We redesigned the survey to allow rating of individual items contributing to the abstract in Round 2 (see download at <https://doi.org/10.15131/shef.data.6198347> for this abstract checklist). We agreed in principle on suggested additions; however, felt we should strike a balance between the content of the abstract and restriction on word limit. Therefore, we focus on critical abstract aspects |
| 2b | Mixed opinions of specificity of the adapted item and unsure if the item is specific to AD trials | We reworded the item and retained it to gather more feedback in Round 2 |
| 3a | Reported some difficulties in understanding the adapted item | We reworded the item for clarification |
| 3b | Query on the necessity of justifying the rationale for the AD when not required for fixed sample size designs in standard CONSORT | We retained the item to gather more feedback in Round 2 |
| 8c | Suggested merging the item with 8b. Given some methodological uncertainties, the need for adequate reporting of adaptive response-randomization aspects was highlighted | We acknowledged feedback and retained the item as standalone only to gather further feedback in Round 2 and merged with text for 8b afterwards |
| 12c -12g | Some concerns expressed about the feasibility of including all aspects of statistical methods | Same response as given for item 4 above. We kept individual items to gather further feedback in round 2. After round 2, reword 12c to capture “statistical methods used for estimation and to make inference” – an indirect way to merge 12c -12g text into 12c |
| 14a | Suggested modification to capture different recruitment periods as a result of a trial adaptation, for instance, when new arms are added or some arms are terminated | We modified the item to ensure it captures differences in recruitment periods across treatment arms |
| 14b |  | We reworded the item for logical clarification |
| 14c | Some confusion expressed about the presentation of 14c, 14d and 14e, but some preferred 14c to 14d and 14e | We were testing preference in presentation so acknowledged feedback. We retained 14c and sought further feedback in Round 2 |
| 17a | Mixed opinions on content and suggestions to either expand or contract checklist item (due to the feasibility of reporting) | Acknowledge feedback, but item content remained unchanged and feedback sought in Round 2 |
| 24b -24f | Some aspects overlap and the statistical analysis plan is a key trial document. Some concern expressed about the feasibility of reporting all items | Same response as given for item 4 above. Retained individual items to gather further feedback in Round 2 then merge 24b - 24f thereafter |

For item description, see download at <https://doi.org/10.15131/shef.data.6198290>.
