# Supplementary material for: Development process of a consensus-driven CONSORT extension for randomised trials using an adaptive design
Source: BMC Med. 2018 Nov 16;16:210. doi: 10.1186/s12916-018-1196-2 (PMC6238302; doi:10.1186/s12916-018-1196-2)
Supplement: Supplementary file 5 — Demographic and characteristics of registered participants and responders. Summaries of Delphi survey participants. (DOCX 23 kb) [file 12916_2018_1196_MOESM5_ESM.docx]

| **Characterisation** | ***Round 1*** | | ***Round 2*** | |
| --- | --- | --- | --- | --- |
|  | Registered | Responders | Registered | Responders |
|  | (N = 143) | (N = 94) | (N=156) | (N=114) |
| **Response rate** | NA | 94/143 (65.7%) | NA | 114/156 (73.1%) |
| **Geographical location** |  |  |  |  |
| Argentina | 1(0.7%) | - | 1(0.6%) | - |
| Australia | 2(1.4%) | 2(2.1%) | 2(1.3%) | 2(1.8%) |
| Austria | 6(4.2%) | 5(5.3%) | 6(3.8%) | 5(4.4%) |
| Belgium | 2(1.4%) | 1(1.1%) | 2(1.3%) | 1(0.9%) |
| Bulgaria | 1(0.7%) | 1(1.1%) | 1(0.6%) | 1(0.9%) |
| Denmark | 1(0.7%) | - | 1(0.6%) | 1(0.9%) |
| Estonia | - | - | 1(0.6%) | 1(0.9%) |
| France | 5(3.5%) | 4(4.3%) | 6(3.8%) | 6(5.3%) |
| Germany | 8(5.6%) | 7(7.4%) | 8(5.1%) | 6(5.3%) |
| India | 1(0.7%) | 1(1.1%) | 1(0.6%) | 1(0.9%) |
| Ireland | 1(0.7%) | - | 1(0.6%) | 1(0.9%) |
| Italy | 2(1.4%) | 1(1.1%) | 2(1.3%) | 2(1.8%) |
| Japan | 2(1.4%) | 2(2.1%) | 2(1.3%) | 2(1.8%) |
| Luxembourg | 1(0.7%) | 1(1.1%) | 1(0.6%) | - |
| Malawi | 1(0.7%) | 1(1.1%) | 1(0.6%) | 1(0.9%) |
| Netherlands | 4(2.8%) | 3(3.2%) | 5(3.2%) | 2(1.8%) |
| Norway | 1(0.7%) | 1(1.1%) | 1(0.6%) | 1(0.9%) |
| Romania | 1(0.7%) | 1(1.1%) | 1(0.6%) | 1(0.9%) |
| South Africa | 1(0.7%) | 1(1.1%) | 1(0.6%) | 1(0.9%) |
| South Korea | 2(1.4%) | 1(1.1%) | 2(1.3%) | 1(0.9%) |
| Spain | 1(0.7%) | - | 1(0.6%) | - |
| Sweden | 2(1.4%) | - | 2(1.3%) | 1(0.9%) |
| Switzerland | 1(0.7%) | 1(1.1%) | 2(1.3%) | - |
| United Kingdom | 65(45.5%) | 43(45.7%) | 72(46.2%) | 50(43.9%) |
| United States of America | 31(21.7%) | 17(18.1%) | 33(21.2%) | 27(23.7%) |
|  |  |  |  |  |
| **Self-identified stakeholder group** |  |  |  |  |
| Clinical trial user | 21(14.7%) | 13(13.8%) | 32(20.5%) | 23(20.2%) |
| Clinical trialist | 64(44.8%) | 42(44.7%) | 65(41.7%) | 48(42.1%) |
| Methodologist | 58(40.6%) | 39(41.5%) | 59(37.8%) | 43(37.7%) |
|  |  |  |  |  |
| **Primary role in trials related research** |  |  |  |  |
| Clinical investigator | 18(12.6%) | 13(13.8%) | 18(11.5%) | 12(10.5%) |
| Health economist | 2(1.4%) | 1(1.1%) | 2(1.3%) | 2(1.8%) |
| Journal editor | 1(0.7%) | - | 11(7.1%) | 8(7.0%) |
| Regulatory assessor | 2(1.4%) | 2(2.1%) | 2(1.3%) | 2(1.8%) |
| Research ethicist | 2(1.4%) | 1(1.1%) | 2(1.3%) | 1(0.9%) |
| Research funding board/panel member | 3(2.1%) | 1(1.1%) | 3(1.9%) | 3(2.6%) |
| Statistician | 90(62.9%) | 62(66.0%) | 93(59.6%) | 67(58.8%) |
| Trial manager | 2(1.4%) | - | 2(1.3%) | 1(0.9%) |
| Trial methodologist | 20(14.0%) | 12(12.8%) | 20(12.8%) | 16(14.0%) |
| Other | 3(2.1%) | 2(2.1%) | 3(1.9%) | 2(1.8%) |
|  |  |  |  |  |
| **Secondary roles in trials research (overlapping)** |  |  |  |  |
| Clinical investigator | 10(7.0%) | 4(4.3%) | 12(7.7%) | 10(8.8%) |
| Health economist | 5(3.5%) | 3(3.2%) | 5(3.2%) | 3(2.6%) |
| Journal editor | 17(11.9%) | 14(14.9%) | 18(11.5%) | 15(13.2%) |
| Regulatory assessor | 6(4.2%) | 4(4.3%) | 6(3.8%) | 3(2.6%) |
| Research ethicist | 6(4.2%) | 4(4.3%) | 7(4.5%) | 6(5.3%) |
| Research funding board/panel member | 21(14.7%) | 15(16.0%) | 22(14.1%) | 18(15.8%) |
| Statistician | 41(28.7%) | 26(27.7%) | 41(26.3%) | 30(26.3%) |
| Trial methodologist | 74(51.7%) | 49(52.1%) | 76(48.7%) | 57(50.0%) |
| Patient representative | 1(0.7%) | 1(1.1%) | 2(1.3%) | 2(1.8%) |
| Systematic reviewer | 16(11.2%) | 12(12.8%) | 18(11.5%) | 12(10.5%) |
| Other | 2(1.4%) | 1(1.1%) | 3(1.9%) | - |
|  |  |  |  |  |
| **Current employment sector** |  |  |  |  |
| Public sector | 101(70.6%) | 66(70.2%) | 104(66.7%) | 78(68.4%) |
| Industry | 36(25.2%) | 22(23.4%) | 47(30.1%) | 31(27.2%) |
| Charity | 5(3.5%) | 4(4.3%) | 5(3.2%) | 4(3.5%) |
| Regulatory agency | 4(2.8%) | 3(3.2%) | 4(2.6%) | 2(1.8%) |
| Other | 4(2.8%) | 3(3.2%) | 4(2.6%) | 3(2.6%) |
|  |  |  |  |  |
| **Adaptive designs related experience** |  |  |  |  |
| Research proposal development including trial design | 112(78.3%) | 76(80.9%) | 116(74.4%) | 87(76.3%) |
| Trial conduct and reporting | 101(70.6%) | 65(69.1%) | 107(68.6%) | 76(66.7%) |
| Peer-reviewing or commissioning of research grant proposals for funding | 70(49.0%) | 42(44.7%) | 72(46.2%) | 53(46.5%) |
| Regulatory assessment | 59(41.3%) | 37(39.4%) | 63(40.4%) | 49(43.0%) |
| Peer-review of clinical trials research outputs | 77(53.8%) | 53(56.4%) | 86(55.1%) | 65(57.0%) |
| Peer-review of methodological research outputs | 75(52.4%) | 47(50.0%) | 79(50.6%) | 59(51.8%) |
| Reviewing evidence from trials | 92(64.3%) | 57(60.6%) | 103(66.0%) | 73(64.0%) |
|  |  |  |  |  |
| **Experience in trials related research (years)** |  |  |  |  |
| Median(IQR) | 13(8, 24) | 16(8, 25) | 13(8, 24) | 13(8, 24) |
| Minimum, maximum | 1, 31 | 1, 31 | 1, 31 | 1, 31 |
|  |  |  |  |  |

IQR, interquartile range (25^th^, 75^th^ percentiles); NA, Not applicable; ‘-’, 0(0.0%)
