# Supplementary material for: Development process of a consensus-driven CONSORT extension for randomised trials using an adaptive design
Source: BMC Med. 2018 Nov 16;16:210. doi: 10.1186/s12916-018-1196-2 (PMC6238302; doi:10.1186/s12916-018-1196-2)
Supplement: Supplementary file 6 — Round 1 summary of perceptions about the importance of reporting items. Summaries of round 1 perceptions about the importance of reporting items. (DOCX 28 kb) [file 12916_2018_1196_MOESM6_ESM.docx]

| **Reporting item** | ***Index*** | **Categorical scale** | | | | | **Rating scale** | | | |
| --- | --- | --- | --- | --- | --- | --- | --- | --- | --- | --- |
|  |  | ***N*** | ***Not important*** | ***Important*** | ***Critical*** | ***Don't know*** | ***N*** | ***Median (IQR)*** | ***Mean (SD)*** | ***Min, max*** |
| (1a) Title identification as randomised trial | U | 94 | - | 17(18.1%) | 77(81.9%) | - | 94 | 8.0(7.0, 9.0) | 7.9(1.3) | 5.0, 9.0 |
| (1b) Structured summary | M | 93 | - | 2(2.2%) | 89(95.7%) | 2(2.2%) | 91 | 8.0(8.0, 9.0) | 8.3(1.0) | 4.0, 9.0 |
| (2a) Scientific background and rationale | U | 93 | 2(2.2%) | 11(11.8%) | 80(86.0%) | - | 93 | 8.0(7.0, 9.0) | 7.7(1.5) | 2.0, 9.0 |
| (2b) Specific objectives and hypotheses | M | 93 | - | 3(3.2%) | 89(95.7%) | 1(1.1%) | 92 | 9.0(8.0, 9.0) | 8.3(0.9) | 5.0, 9.0 |
| (3a) Description of the trial design | M | 91 | - | 3(3.3%) | 88(96.7%) | - | 91 | 9.0(8.0, 9.0) | 8.4(0.8) | 5.0, 9.0 |
| (3b) Rationale for an adaptive design | N | 91 | 2(2.2%) | 16(17.6%) | 73(80.2%) | - | 91 | 8.0(7.0, 9.0) | 7.6(1.5) | 2.0, 9.0 |
| (3c) Specification of pre-planned adaptation | N | 91 | - | 7(7.7%) | 83(91.2%) | 1(1.1%) | 90 | 9.0(8.0, 9.0) | 8.2(1.1) | 4.0, 9.0 |
| (3d) Unplanned changes to the trial design or methods | M | 91 | - | 7(7.7%) | 83(91.2%) | 1(1.1%) | 90 | 8.0(8.0, 9.0) | 8.1(1.1) | 4.0, 9.0 |
| (3e) Adaptive design properties | N | 91 | 2(2.2%) | 21(23.1%) | 68(74.7%) | - | 91 | 8.0(6.0, 9.0) | 7.5(1.6) | 2.0, 9.0 |
| (4a) Participants eligibility | U | 90 | 1(1.1%) | 13(14.4%) | 76(84.4%) | - | 90 | 8.0(7.0, 9.0) | 7.7(1.5) | 3.0, 9.0 |
| (4b) Setting | U | 90 | 5(5.6%) | 32(35.6%) | 53(58.9%) | - | 90 | 7.0(5.0, 9.0) | 6.8(1.9) | 20, 9.0 |
| (5) Intervention | U | 90 | 1(1.1%) | 12(13.3%) | 77(85.6%) | - | 90 | 8.0(7.0, 9.0) | 7.8(1.4) | 3.0, 9.0 |
| (6a) Pre-specified outcomes | M | 90 | - | 6(6.7%) | 84(93.3%) | - | 90 | 9.0(8.0, 9.0) | 8.2(1.0) | 4.0, 9.0 |
| (6b) Unplanned changes to outcomes | M | 90 | - | 14(15.6%) | 76(84.4%) | - | 90 | 8.0(7.0, 9.0) | 7.9(1.2) | 4.0, 9.0 |
| (7a) Sample size | M | 90 | 1(1.1%) | 13(14.4%) | 76(84.4%) | - | 90 | 8.0(7.0, 9.0) | 7.8(1.4) | 3.0, 9.0 |
| (7b) Decision-making criteria to guide adaptation | N | 90 | 3(3.3%) | 6(6.7%) | 80(88.9%) | 1(1.1%) | 89 | 8.0(8.0, 9.0) | 8.0(1.5) | 2.0, 9.0 |
| (8a) Method used to generate randomisation sequence | U | 89 | 10(11.2%) | 30(33.7%) | 49(55.1%) | - | 89 | 7.0(5.0, 9.0) | 6.6(2.2) | 1.0, 9.0 |
| (8b) Type of randomisation | U | 89 | 4(4.5%) | 29(32.6%) | 56(62.9%) | - | 89 | 7.0(6.0, 9.0) | 7.0(1.8) | 3.0, 9.0 |
| (8c) Randomisation updates after trial commencement | N | 89 | 3(3.4%) | 17(19.1%) | 69(77.5%) | - | 89 | 8.0(7.0, 9.0) | 7.6(1.6) | 2.0, 9.0 |
| (9) Allocation concealment mechanism | U | 89 | 8(9.0%) | 30(33.7%) | 51(57.3%) | - | 89 | 7.0(5.0, 9.0) | 6.6(2.1) | 1.0, 9.0 |
| (10) Who generated the random allocation sequence | U | 89 | 17(19.1%) | 33(37.1%) | 39(43.8%) | - | 89 | 6.0(4.0, 8.0) | 6.0(2.4) | 1.0, 9.0 |
| (11a) Who was blinded | U | 89 | 2(2.2%) | 20(22.5%) | 67(75.3%) | - | 89 | 8.0(7.0, 9.0) | 7.5(1.7) | 1.0, 9.0 |
| (11b) Interventions similarity | U | 89 | 14(15.7%) | 35(39.3%) | 34(38.2%) | 6(6.7%) | 83 | 6.0(4.0, 8.0) | 6.0(2.4) | 1.0, 9.0 |
| (11c) Confidentiality & minimisation of operational bias | N | 89 | 4(4.5%) | 28(31.5%) | 57(64.0%) | - | 89 | 7.0(6.0, 9.0) | 7.1(1.8) | 1.0, 9.0 |
| (12a) Statistical methods used to compare groups | M | 89 | 1(1.1%) | 2(2.2%) | 86(96.6%) | - | 89 | 9.0(8.0, 9.0) | 8.3(1.0) | 3.0, 9.0 |
| (12b) Methods for additional analyses | U | 89 | - | 28(31.5%) | 61(68.5%) | - | 89 | 8.0(6.0, 9.0) | 7.3(1.5) | 4.0, 9.0 |
| (12c) Inferential methods/procedures | N | 89 | - | 10(11.2%) | 77(86.5%) | 2(2.2%) | 87 | 8.0(7.0, 9.0) | 8.0(1.1) | 4.0, 9.0 |
| (12d) Methods to combine data across interim stages | N | 89 | - | 13(14.6%) | 73(82.0%) | 3(3.4%) | 86 | 8.0(7.0, 9.0) | 7.7(1.3) | 4.0, 9.0 |
| (12e) Dealing with overrun participants | N | 89 | 2(2.2%) | 30(33.7%) | 54(60.7%) | 3(3.4%) | 86 | 7.0(6.0, 9.0) | 7.0(1.7) | 2.0, 9.0 |
| (12f) Dealing with multiple outcomes/multiple treatments | N | 89 | - | 10(11.2%) | 78(87.6%) | 1(1.1%) | 88 | 8.0(7.0, 9.0) | 7.9(1.2) | 4.0, 9.0 |
| (12g) Prior selection | N | 89 | 3(3.4%) | 24(27.0%) | 58(65.2%) | 4(4.5%) | 85 | 8.0(6.0, 9.0) | 7.3(1.8) | 2.0, 9.0 |
| (13a) Randomised, received intended treatment ... | M | 89 | - | 14(15.7%) | 74(83.1%) | 1(1.1%) | 88 | 8.0(7.0, 9.0) | 7.9(1.3) | 4.0, 9.0 |
| (13b) Losses and exclusions after randomisation | M | 89 | 1(1.1%) | 17(19.1%) | 71(79.8%) | - | 89 | 8.0(7.0, 9.0) | 7.7(1.5) | 3.0, 9.0 |
| (14a) Dates defining the period of recruitment | U | 89 | 9(10.1%) | 29(32.6%) | 51(57.3%) | - | 89 | 7.0(5.0, 8.0) | 6.6(2.1) | 2.0, 9.0 |
| (14b) Unexpected termination | U | 89 | - | 9(10.1%) | 80(89.9%) | - | 89 | 9.0(7.0, 9.0) | 8.1(1.2) | 4.0, 9.0 |
| (14c) Adaptation decisions (planned and unplanned) | N | 89 | 2(2.2%) | 7(7.9%) | 78(87.6%) | 2(2.2%) | 87 | 8.0(7.0, 9.0) | 8.0(1.6) | 1.0, 9.0 |
| (14d) Pre-planned adaptation decisions | N | 89 | 4(4.5%) | 7(7.9%) | 77(86.5%) | 1(1.1%) | 88 | 8.0(7.0, 9.0) | 7.8(1.8) | 1.0, 9.0 |
| (14e) Deviations from pre-planned decisions | N | 89 | 3(3.4%) | 10(11.2%) | 73(82.0%) | 3(3.4%) | 86 | 8.0(7.0, 9.0) | 7.7(1.8) | 1.0, 9.0 |
| (15a) Appropriate baseline data for comparability | M | 89 | 3(3.4%) | 29(32.6%) | 56(62.9%) | 1(1.1%) | 88 | 7.5(6.0, 9.0) | 7.2(1.8) | 1.0, 9.0 |
| (15b) Representativeness of patient population | N | 89 | 6(6.7%) | 36(40.4%) | 47(52.8%) | - | 89 | 7.0(5.0, 8.0) | 6.6(1.8) | 3.0, 9.0 |
| (16) Numbers analysed at the interim & final analysis | M | 89 | - | 8(9.0%) | 81(91.0%) | - | 89 | 8.0(7.0, 9.0) | 8.0(1.2) | 4.0, 9.0 |
| (17a) Primary outcome results | M | 87 | 1(1.1%) | 8(9.2%) | 78(89.7%) | - | 87 | 9.0(8.0, 9.0) | 8.2(1.2) | 3.0, 9.0 |
| (17b) Presentation of binary outcomes | U | 87 | 6(6.9%) | 26(29.9%) | 52(59.8%) | 3(3.4%) | 84 | 7.0(6.0, 8.0) | 6.8(1.9) | 1.0, 9.0 |
| (17c) Suitable representation of interim results | N | 87 | 1(1.1%) | 26(29.9%) | 60(69.0%) | - | 87 | 8.0(6.0, 9.0) | 7.3(1.5) | 3.0, 9.0 |
| (18) Ancillary analyses | U | 87 | 3(3.4%) | 39(44.8%) | 43(49.4%) | 2(2.3%) | 85 | 7.0(5.0, 8.0) | 6.4(1.8) | 1.0, 9.0 |
| (19) Important harms & unintended effects | U | 86 | 2(2.3%) | 14(16.3%) | 69(80.2%) | 1(1.2%) | 85 | 8.0(7.0, 9.0) | 7.8(1.6) | 1.0, 9.0 |
| (20) Limitations, sources of bias, imprecision &deviations | M | 86 | - | 11(12.8%) | 75(87.2%) | - | 86 | 8.0(7.0, 9.0) | 7.8(1.2) | 4.0, 9.0 |
| (21) Generalisability - external validity & applicability | M | 86 | 1(1.2%) | 18(20.9%) | 67(77.9%) | - | 86 | 8.0(7.0, 9.0) | 7.5(1.4) | 3.0, 9.0 |
| (22a) Interpretation consistent with results | U | 86 | - | 18(20.9%) | 68(79.1%) | - | 86 | 8.0(7.0, 9.0) | 7.6(1.3) | 4.0, 9.0 |
| (22b) Contribution to future related research | N | 86 | 13(15.1%) | 43(50.0%) | 30(34.9%) | - | 86 | 6.0(5.0, 7.0) | 5.8(1.9) | 1.0, 9.0 |
| (23) Trial registration | U | 86 | 7(8.1%) | 21(24.4%) | 58(67.4%) | - | 86 | 8.0(6.0, 9.0) | 7.1(2.2) | 1.0, 9.0 |
| (24a) Trial protocol | U | 86 | 5(5.8%) | 20(23.3%) | 61(70.9%) | - | 86 | 8.0(6.0, 9.0) | 7.3(1.9) | 2.0, 9.0 |
| (24b) Intentionally withheld information | N | 86 | 8(9.3%) | 30(34.9%) | 42(48.8%) | 6(7.0%) | 80 | 7.0(5.0, 8.0) | 6.4(2.1) | 1.0, 9.0 |
| (24c) Statistical analysis plan | N | 86 | 6(7.0%) | 34(39.5%) | 44(51.2%) | 2(2.3%) | 84 | 7.0(5.0, 8.0) | 6.5(1.9) | 1.0, 9.0 |
| (24d) Simulation protocol and report | N | 86 | 10(11.6%) | 35(40.7%) | 38(44.2%) | 3(3.5%) | 83 | 6.0(5.0, 8.0) | 6.2(2.0) | 1.0, 9.0 |
| (24e) (Independent) Data Monitoring Committee Charter | N | 86 | 15(17.4%) | 32(37.2%) | 38(44.2%) | 1(1.2%) | 85 | 6.0(4.0, 7.0) | 5.9(2.1) | 1.0, 9.0 |
| (24f) Statistical code | N | 86 | 18(20.9%) | 41(47.7%) | 25(29.1%) | 2(2.3%) | 84 | 5.0(4.0, 7.0) | 5.4(2.1) | 1.0, 9.0 |
| (25) Sources of funding and other support | U | 86 | 7(8.1%) | 23(26.7%) | 56(65.1%) | - | 86 | 8.0(6.0, 9.0) | 7.0(2.2) | 1.0, 9.0 |
|  |  |  |  |  |  |  |  |  |  |  |

Index: N, new item; M, modified item; U, unchanged item (retained as they appear in the CONSORT 2010 checklist); IQR, interquartile range (25^th^, 75^th^ percentiles); min, minimum; max, maximum; SD, standard deviation; ‘-’, 0(0.0%); for the item description, see download at <https://doi.org/10.15131/shef.data.6198290>.
