# Supplementary material for: Development process of a consensus-driven CONSORT extension for randomised trials using an adaptive design
Source: BMC Med. 2018 Nov 16;16:210. doi: 10.1186/s12916-018-1196-2 (PMC6238302; doi:10.1186/s12916-018-1196-2)
Supplement: Supplementary file 7 — Round 2 summary of perceptions about the importance of reporting items. Summaries of round 2 perceptions about the importance of reporting items. (DOCX 29 kb) [file 12916_2018_1196_MOESM7_ESM.docx]

| **Reporting item (new or modified)** |  | **Categorical scale** | | | | | **Rating scale** | | | |
| --- | --- | --- | --- | --- | --- | --- | --- | --- | --- | --- |
|  | ***Index*** | ***N*** | ***Not important*** | ***Important*** | ***Critical*** | ***Don't know*** | ***N*** | ***Median(IQR)*** | ***Mean(SD)*** | ***Min, max*** |
| **Abstract checklist** |  |  |  |  |  |  |  |  |  |  |
| (1b) Description of the trial design (abstract) | M | 114 | 1(0.9%) | 9(7.9%) | 104(91.2%) | - | 114 | 9.0(8.0, 9.0) | 8.2(1.2) | 2.0, 9.0 |
| (1c) Clearly defined primary outcome for this report | M | 114 | 1(0.9%) | 8(7.0%) | 105(92.1%) | - | 114 | 8.0(7.0, 9.0) | 8.1(1.1) | 3.0, 9.0 |
| (1d) Result for each group, effect size & its precision | M | 114 | 5(4.4%) | 24(21.1%) | 85(74.6%) | - | 114 | 8.0(6.0, 9.0) | 7.5(1.7) | 1.0, 9.0 |
| (1e) Adaptation decisions made | N | 114 | 2(1.8%) | 37(32.5%) | 75(65.8%) | - | 114 | 7.0(6.0, 8.0) | 7.1(1.6) | 1.0, 9.0 |
| (1f) Conclusions | M | 114 | 1(0.9%) | 22(19.3%) | 90(78.9%) | 1(0.9%) | 113 | 8.0(7.0, 9.0) | 7.6(1.5) | 2.0, 9.0 |
| **Main checklist** |  |  |  |  |  |  |  |  |  |  |
| (3a) Description of the trial design | M | 112 | - | 3(2.7%) | 107(95.5%) | 2(1.8%) | 110 | 9.0(9.0, 9.0) | 8.7(0.7) | 5.0, 9.0 |
| (3b) Rationale for an adaptive design | N | 112 | 1(0.9%) | 8(7.1%) | 103(92.0%) | - | 112 | 8.0(7.0, 9.0) | 8.0(1.2) | 3.0, 9.0 |
| (3c) Specification of pre-planned adaptation | N | 112 | 1(0.9%) | 4(3.6%) | 106(94.6%) | 1(0.9%) | 111 | 9.0(8.0, 9.0) | 8.4(1.0) | 3.0, 9.0 |
| (3d) Unplanned changes to the trial design or methods | M | 112 | 1(0.9%) | 4(3.6%) | 105(93.8%) | 2(1.8%) | 110 | 9.0(8.0, 9.0) | 8.3(1.1) | 1.0, 9.0 |
| (3e) Adaptive design properties | N | 112 | 1(0.9%) | 12(10.7%) | 98(87.5%) | 1(0.9%) | 111 | 8.0(8.0, 9.0) | 7.9(1.3) | 3.0, 9.0 |
| (6a) Pre-specified outcomes | M | 112 | - | 3(2.7%) | 109(97.3%) | - | 112 | 9.0(8.0, 9.0) | 8.6(0.7) | 6.0, 9.0 |
| (6b) Unplanned changes to outcomes | M | 112 | 1(0.9%) | 4(3.6%) | 106(94.6%) | 1(0.9%) | 111 | 9.0(8.0, 9.0) | 8.4(1.1) | 2.0, 9.0 |
| (7a) Sample size | M | 112 | - | 6(5.4%) | 104(92.9%) | 2(1.8%) | 110 | 9.0(8.0, 9.0) | 8.2(1.0) | 5.0, 9.0 |
| (7b) Decision-making criteria to guide adaptation | N | 112 | - | 4(3.6%) | 107(95.5%) | 1(0.9%) | 111 | 9.0(8.0, 9.0) | 8.4(0.9) | 4.0, 9.0 |
| (8c) Randomisation updates after trial commencement | N | 111 | - | 16(14.4%) | 94(84.7%) | 1(0.9%) | 110 | 8.0(7.0, 9.0) | 7.9(1.3) | 4.0, 9.0 |
| (11c) Confidentiality & minimisation of operational bias | N | 111 | 1(0.9%) | 28(25.2%) | 82(73.9%) | - | 111 | 7.0(6.0, 8.0) | 7.3(1.3) | 1.0, 9.0 |
| (12a) Statistical methods used to compare groups | M | 111 | - | 2(1.8%) | 108(97.3%) | 1(0.9%) | 110 | 9.0(9.0, 9.0) | 8.8(0.6) | 6.0, 9.0 |
| (12c) Inferential methods/procedures | N | 111 | - | 6(5.4%) | 103(92.8%) | 2(1.8%) | 109 | 9.0(8.0, 9.0) | 8.5(0.8) | 5.0, 9.0 |
| (12d) Methods to combine data across interim stages | N | 111 | 1(0.9%) | 6(5.4%) | 101(91.0%) | 3(2.7%) | 108 | 8.0(8.0, 9.0) | 8.1(1.2) | 1.0, 9.0 |
| (12e) Dealing with overrun participants | N | 111 | 1(0.9%) | 38(34.2%) | 69(62.2%) | 3(2.7%) | 108 | 7.0(6.0, 8.0) | 7.1(1.3) | 3.0, 9.0 |
| (12f) Dealing with multiple outcomes/multiple treatments | N | 111 | - | 6(5.4%) | 104(93.7%) | 1(0.9%) | 110 | 8.0(8.0, 9.0) | 8.1(1.0) | 4.0, 9.0 |
| (12g) Prior selection | N | 111 | 4(3.6%) | 24(21.6%) | 82(73.9%) | 1(0.9%) | 110 | 8.0(6.0, 9.0) | 7.4(1.7) | 2.0, 9.0 |
| (13a) Randomised, received intended treatment ... | M | 110 | - | 6(5.5%) | 103(93.6%) | 1(0.9%) | 109 | 9.0(8.0, 9.0) | 8.4(1.0) | 4.0, 9.0 |
| (14a) Dates defining the period of recruitment | M | 110 | 4(3.6%) | 23(20.9%) | 82(74.5%) | 1(0.9%) | 109 | 7.0(7.0, 9.0) | 7.2(1.6) | 2.0, 9.0 |
| (14b) Unexpected termination | M | 110 | - | 7(6.4%) | 101(91.8%) | 2(1.8%) | 108 | 9.0(9.0, 9.0) | 8.6(1.1) | 4.0, 9.0 |
| (14c) Adaptation decisions (planned and unplanned) | N | 110 | - | 4(3.6%) | 103(93.6%) | 3(2.7%) | 107 | 9.0(8.0, 9.0) | 8.5(0.9) | 4.0, 9.0 |
| (15a) Appropriate baseline data for comparability | M | 110 | 4(3.6%) | 23(20.9%) | 83(75.5%) | - | 110 | 8.0(7.0, 9.0) | 7.5(1.6) | 2.0, 9.0 |
| (15b) Representativeness of patient population | N | 110 | 3(2.7%) | 41(37.3%) | 66(60.0%) | - | 110 | 7.0(6.0, 8.0) | 6.8(1.4) | 2.0, 9.0 |
| (16) Numbers analysed at the interim & final analysis | M | 110 | - | 5(4.5%) | 104(94.5%) | 1(0.9%) | 109 | 9.0(8.0, 9.0) | 8.5(0.9) | 5.0, 9.0 |
| (17a) Primary outcome results | M | 110 | 1(0.9%) | 5(4.5%) | 101(91.8%) | 3(2.7%) | 107 | 9.0(8.0, 9.0) | 8.5(1.0) | 3.0, 9.0 |
| (17c) Suitable representation of interim outcome results | N | 110 | - | 22(20.0%) | 87(79.1%) | 1(0.9%) | 109 | 8.0(7.0, 8.0) | 7.6(1.3) | 4.0, 9.0 |
| (20) Limitations, sources of bias, imprecision & deviations | M | 110 | - | 3(2.7%) | 106(96.4%) | 1(0.9%) | 109 | 8.0(8.0, 9.0) | 8.3(0.9) | 4.0, 9.0 |
| (22b) Contribution to future related research | N | 110 | 15(13.6%) | 62(56.4%) | 33(30.0%) | - | 110 | 6.0(5.0, 7.0) | 5.6(1.8) | 1.0, 9.0 |
| (24b) Intentionally withheld information | N | 110 | 7(6.4%) | 36(32.7%) | 66(60.0%) | 1(0.9%) | 109 | 7.0(6.0, 8.0) | 6.7(1.8) | 2.0, 9.0 |
| (24c) Statistical analysis plan | N | 110 | 4(3.6%) | 31(28.2%) | 74(67.3%) | 1(0.9%) | 109 | 7.0(6.0, 8.0) | 7.0(1.6) | 1.0, 9.0 |
| (24d) Simulation protocol and report | N | 110 | 6(5.5%) | 53(48.2%) | 49(44.5%) | 2(1.8%) | 108 | 6.0(5.0, 7.5) | 6.4(1.7) | 1.0, 9.0 |
| (24e) Data Monitoring Committee Charter | N | 110 | 15(13.6%) | 50(45.5%) | 44(40.0%) | 1(0.9%) | 109 | 6.0(5.0, 7.0) | 5.8(1.8) | 1.0, 9.0 |
| (24f) Statistical code | N | 110 | 21(19.1%) | 58(52.7%) | 29(26.4%) | 2(1.8%) | 108 | 5.0(4.0, 7.0) | 5.4(2.0) | 1.0, 9.0 |
|  |  |  |  |  |  |  |  |  |  |  |

Index: N, new item; M, modified item; IQR, interquartile range (25^th^, 75^th^ percentiles); min, minimum; max, maximum; SD, standard deviation; ‘-’, 0(0.0%); for a detailed description of the item, see download at <https://doi.org/10.15131/shef.data.6198347>
