# Supplementary material for: Development process of a consensus-driven CONSORT extension for randomised trials using an adaptive design
Source: BMC Med. 2018 Nov 16;16:210. doi: 10.1186/s12916-018-1196-2 (PMC6238302; doi:10.1186/s12916-018-1196-2)
Supplement: Supplementary file 8 — Measures of agreement in rating scores between Delphi rounds 1 and 2 survey. Summaries of measures of agreement in the rating of participants between rounds 1 and 2 of the Delphi surveys. (DOCX 24 kb) [file 12916_2018_1196_MOESM8_ESM.docx]

| **Reporting item (new or modified)** | **Perfect agreement** | | **Weighted Kappa** | **Verdict** |
| --- | --- | --- | --- | --- |
|  | *n/N* | *proportion (95%CI)* | *coefficient (95%CI)* |  |
| (3a) Description of the trial design | 45/76 | 59.2% (46.1, 69.7) | 0.19 (0.03, 0.36) | SA |
| (3b) Rationale for an adaptive design | 43/77 | 55.8% (44.2, 66.2) | 0.39 (0.24, 0.54) | FA |
| (3c) Specification of pre-planned adaptation | 41/75 | 54.7% (41.3, 64.0) | 0.25 (0.09, 0.42) | FA |
| (3d) Unplanned changes to the trial design or methods | 38/74 | 51.4% (41.9, 63.5) | 0.24 (0.08, 0.41) | FA |
| (3e) Adaptive design properties | 39/76 | 51.3% (38.2, 61.8) | 0.34 (0.20, 0.49) | FA |
| (6a) Pre-specified outcomes | 46/76 | 60.5% (48.7, 69.7) | 0.31 (0.16, 0.46) | FA |
| (6b) Unplanned changes to outcomes | 44/75 | 58.7% (48.0, 70.7) | 0.34 (0.18, 0.51) | FA |
| (7a) Sample size | 40/74 | 54.1% (44.6, 66.2) | 0.36 (0.21, 0.50) | FA |
| (7b) Decision-making criteria to guide adaptation | 43/74 | 58.1% (44.6, 67.6) | 0.33 (0.18, 0.49) | FA |
| (8c) Randomisation updates after trial commencement | 36/74 | 48.6% (36.5, 58.1) | 0.30 (0.17, 0.45) | FA |
| (11c) Confidentiality & minimisation of operational bias | 39/75 | 52.0% (42.7, 65.3) | 0.40 (0.28, 0.54) | FA |
| (12a) Statistical methods used to compare groups | 44/74 | 59.5% (45.9, 68.9) | 0.20 (0.06, 0.35) | SA |
| (12c) Inferential methods/procedures | 43/73 | 58.9% (46.6, 68.5) | 0.35 (0.22, 0.50) | FA |
| (12d) Methods to combine data across interim stages | 36/71 | 50.7% (40.8, 64.8) | 0.30 (0.17, 0.46) | FA |
| (12e) Dealing with overrun participants | 34/70 | 48.6% (35.7, 58.6) | 0.36 (0.21, 0.51) | FA |
| (12f) Dealing with multiple outcomes/multiple treatments | 41/73 | 56.2% (43.8, 65.8) | 0.37 (0.22, 0.53) | FA |
| (12g) Prior selection | 40/70 | 57.1% (44.3, 67.1) | 0.44 (0.31, 0.58) | MA |
| (13a) Randomised, received intended treatment ... | 41/73 | 56.2% (43.8, 65.8) | 0.31 (0.15, 0.48) | FA |
| (14a) Dates defining the period of recruitment | 29/75 | 38.7% (29.3, 52.0) | 0.26 (0.14, 0.39) | FA |
| (14b) Unexpected termination | 45/73 | 61.6% (52.1, 74.0) | 0.25 (0.13, 0.40) | FA |
| (14c) Adaptation decisions (planned and unplanned) | 34/71 | 47.9% (35.2, 57.7) | 0.15 (0.00, 0.31) | SA |
| (15a) Appropriate baseline data for comparability | 41/74 | 55.4% (41.9, 66.2) | 0.44 (0.29, 0.58) | MA |
| (15b) Representativeness of patient population | 28/75 | 37.3% (24.0, 46.7) | 0.25 (0.11, 0.38) | FA |
| (16) Numbers analysed at the interim & final analysis | 42/74 | 56.8% (43.2, 66.2) | 0.29 (0.14, 0.42) | FA |
| (17a) Primary outcome results | 43/71 | 60.6% (50.7, 71.8) | 0.27 (0.12, 0.44) | FA |
| (17c) Suitable representation of interim outcome results | 34/72 | 47.2% (37.5, 59.7) | 0.32 (0.18, 0.46) | FA |
| (20) Limitations, sources of bias, imprecision &deviations | 40/71 | 56.3% (43.7, 64.8) | 0.35 (0.22, 0.49) | FA |
| (22b) Contribution to future related research | 29/72 | 40.3% (27.8, 51.4) | 0.29 (0.16, 0.45) | FA |
| (24b) Intentionally withheld information | 27/66 | 40.9% (30.3, 54.5) | 0.28 (0.14, 0.43) | FA |
| (24c) Statistical analysis plan | 36/69 | 52.2% (40.6, 65.2) | 0.42 (0.28, 0.56) | MA |
| (24d) Simulation protocol and report | 33/68 | 48.5% (38.2, 60.3) | 0.38 (0.25, 0.52) | FA |
| (24e) Data Monitoring Committee Charter | 28/70 | 40.0% (27.1, 50.0) | 0.30 (0.18, 0.44) | FA |
| (24f) Statistical code | 34/69 | 49.3% (37.7, 60.9) | 0.41 (0.28, 0.56) | MA |
|  |  |  |  |  |

FA, fair agreement; MA, moderate agreement; SA, slight agreement; Perfect agreement is the proportion of responders who did not change their rating scores between rounds
