# Supplementary material for: Development process of a consensus-driven CONSORT extension for randomised trials using an adaptive design
Source: BMC Med. 2018 Nov 16;16:210. doi: 10.1186/s12916-018-1196-2 (PMC6238302; doi:10.1186/s12916-018-1196-2)
Supplement: Supplementary file 10 — Accessible supplementary material hosted within the University of Sheffield ORDA repository. Summary reports; draft checklists used in round 1 and 2 Delphi surveys; registration and Delphi survey rounds datasets; Figures (clustered boxplots) displaying responders’ perceptions of reporting items stratified by key characteristics. (DOCX 22 kb) [file 12916_2018_1196_MOESM10_ESM.docx]

| **Description of files** | **ORDA repository link** |
| --- | --- |
| Summary reports   - First steering committee meeting to generate potential reporting items for the guideline - Qualitative feedback from Round 1 Delphi survey - Qualitative feedback from Round 2 Delphi survey | <https://doi.org/10.15131/shef.data.6139631> |
| Finalised draft checklist used in Round 1 Delphi Survey | <https://doi.org/10.15131/shef.data.6198290> |
| Finalised draft checklist used in Round 2 Delphi Survey (abstract and main report) | <https://doi.org/10.15131/shef.data.6198347> |
| Datasets   - Registration data prior to Round 1 Delphi survey - Additional registration data between Rounds - Round 1 Delphi survey responses - Round 2 Delphi survey responses - Registration data variable description | <https://doi.org/10.15131/shef.data.6120572> |
| Zipped files of clustered boxplots of responders’ perceptions of the importance of reporting items for Round 1 and 2 Delphi surveys stratified by key characteristics (stakeholder group, current employment sector, regulatory assessment experience, and primary role as a statistician) | <https://doi.org/10.15131/shef.data.6139721.v1> |
