# Supplementary material for: Development process of a consensus-driven CONSORT extension for randomised trials using an adaptive design
Source: BMC Med. 2018 Nov 16;16:210. doi: 10.1186/s12916-018-1196-2 (PMC6238302; doi:10.1186/s12916-018-1196-2)
Supplement: Supplementary file 11 — Registered participants for the Delphi surveys. List of participants who registered to take part in the Delphi surveys. This includes only those who did not opt out to be publicly acknowledged. (DOCX 19 kb) [file 12916_2018_1196_MOESM11_ESM.docx]

Susan S. Ellenberg, Susan Halabi, Roger J. Lewis, Janet Wittes, Thomas Friedrich Jaki, Kerry Hood, Caitlyn Ellerbe, Paula Williamson, Robert Silbergleit, James Bolognese, Lisa Hampson, Laura Richert, Carl Jacobus Lombard, Laura Flight, Philipp Aichinger, Kevin Kunzmann, Dennis Görlich, Robert Hill, Richardus Vonk, Christopher Weir, Robert Hills, Jon Nicholl, Cindy Cooper, Donald Stein, Kerry Leask, Kaspar Rufibach, Alan Phillips, Achim Steup, Michael O'Kelly, Alex Freeman, Peter M. van de Ven, Mark Pitt, Timothy C. Guetterman, Ravindra L Mehta, Alessandro Laviano, Martin Jenkins, Toby Richards, Valerie Durkalski, Michael Cole, Inge Christoffer Olsen, Jordan J. Elm, Yves Schymura, Marian Knight, Mairead North, Julien Tanniou, Katie Biggs, Per Broberg, Caitlyn Ellerbe, Rajat Mukherjee, Eric Derobert, Josephine Wolfram, Elodie Blondiaux, Tje Lin Chung, Juergen Zezula, Jusun Nam, Sharon Love, Jane Holmes, Munya Dimairo, Adrian Mander, Tracy Pellatt-Higgins, Victoria Cornelius, Susanne Urach, Sorana D. Bolboacă, Agnieszka Butwicka, Thomas A. Louis, Rui (Sammi) Tang, Graeme MacLennan, Greg Cicconetti, William J. Meurer, Luca Bertolaccini, Stephane Heritier, Ashwani Kumar Mishra, Jørgen Hilden, Matthew Sydes, Cyrus R Mehta, Naohiro Yonemoto, Yinpu Chen, Sammy Yuan, Silke Jörgens, Philip Pallmann, Sofia S. Villar, Chris Harbron, Krassimir Boytchev Kalinov, Franz König, Thomas Nichols, Catey Bunce, Rod Taylor, Janet Dunn, Marianna Mitratza, Frank Waldron-Lynch, Nick Parsons, Judith Bliss, Pouya Alaghband, Richard Adams, Simon Bond, Richard Parker, Olivier Collignon, Ivan Koychev, David Lawrence, Louise M Wallace, William Barsan, David J. Vanness, John Kirkpatrick, Stefan Englert, Yannis Jemiai, Jose Ignacio Valenzuela, Byron J. Gajewski, Julie Ann Marsh, James Matcham, Lisa Douet, Karen C. Johnston, Aiyi Liu, Susan Dutton, Alun Bedding, Brian Hobbs, Rod Taylor, Richard Kay, Mark Pilling, Thomas Zwingers, Murielle Mauer, Dena Howard, Melina Dritsaki, Seung-Ho Kang, Steven Julious, Toshimitsu Hamasaki, Christina Yap, Nicolas Meyer, Telba Irony, David Robertson, Charlotte Leigh, Rachel Marshall, Joan Marsh, Elizabeth Loder, Sophie Cook, Roland Marion-Gallois, José Merino, Wim Weber, Laurent Quinquis, and Jaak Sõnajalg
