# Supplementary material for: Development process of a consensus-driven CONSORT extension for randomised trials using an adaptive design
Source: BMC Med. 2018 Nov 16;16:210. doi: 10.1186/s12916-018-1196-2 (PMC6238302; doi:10.1186/s12916-018-1196-2)

Additional file 9: Distributions of change in ratings from round 1 and Bland-Altman plots

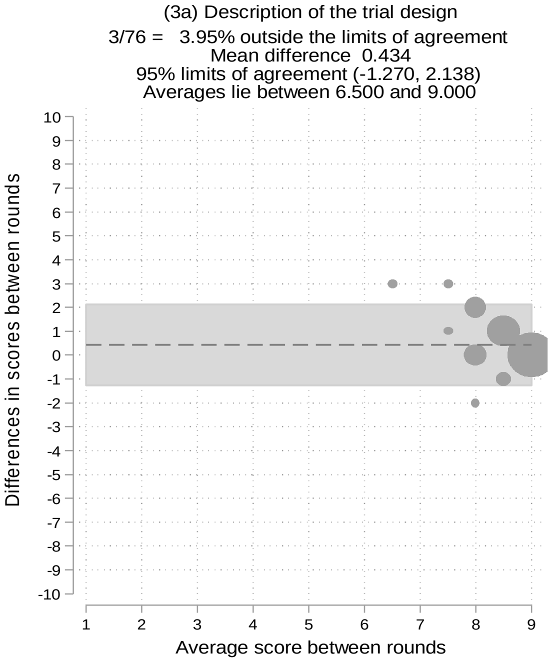

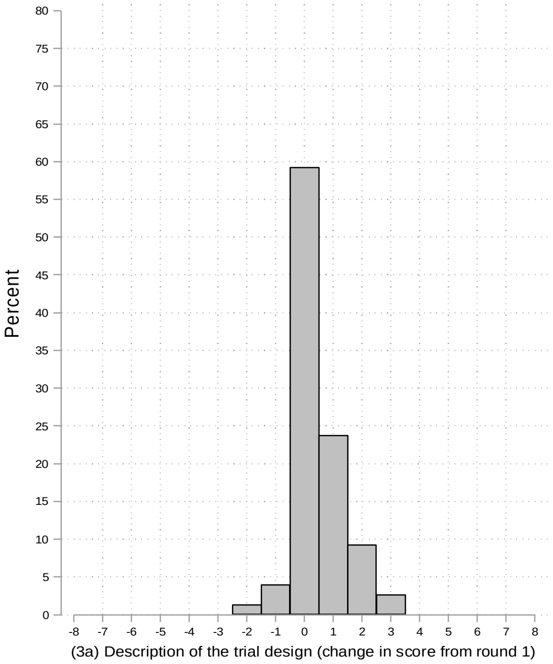

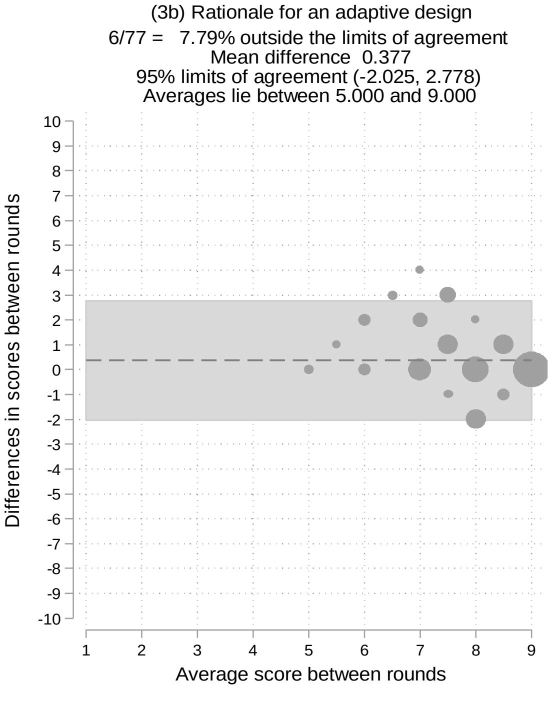

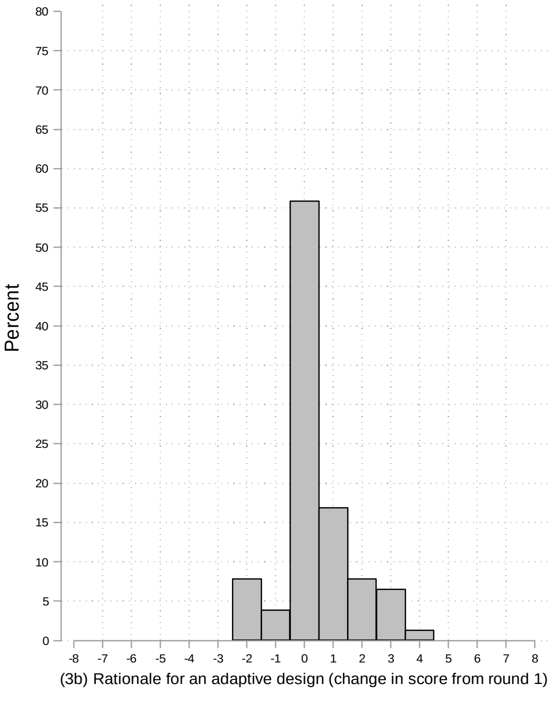

Additional file 9: Distributions of change in ratings from round 1 and Bland-Altman plots

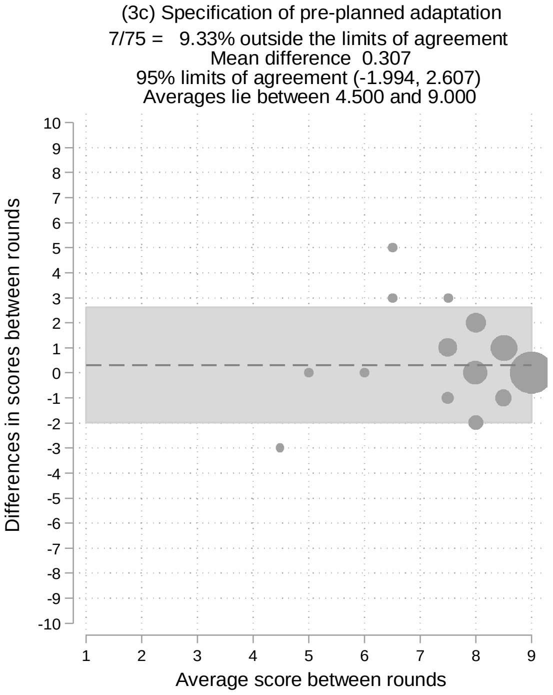

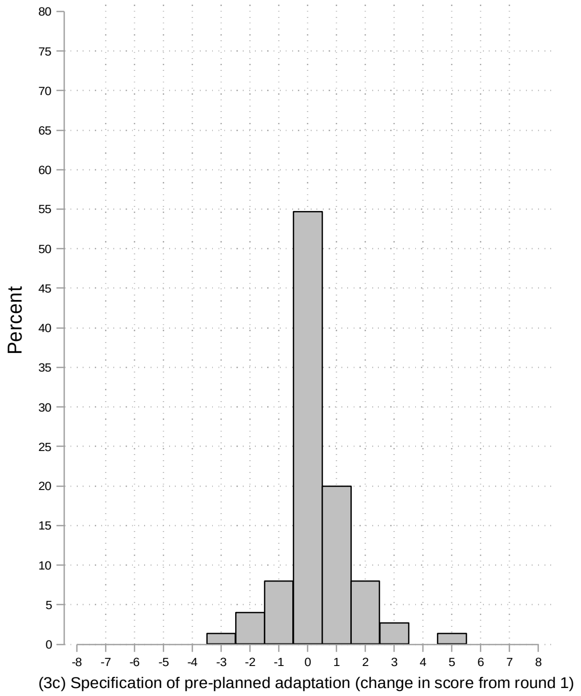

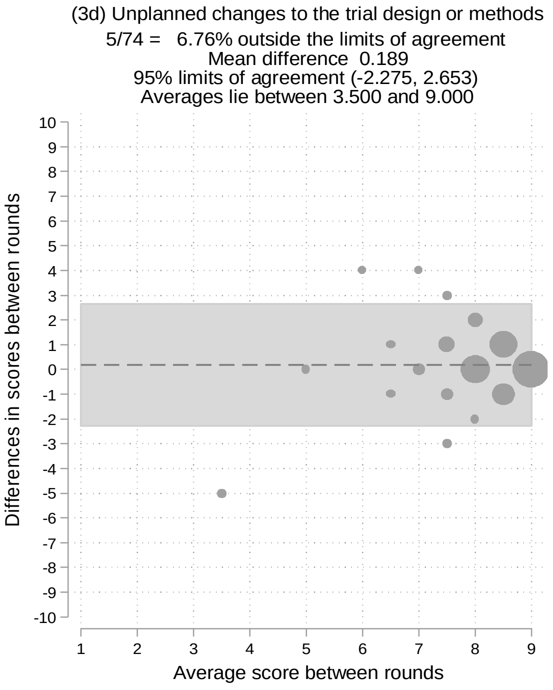

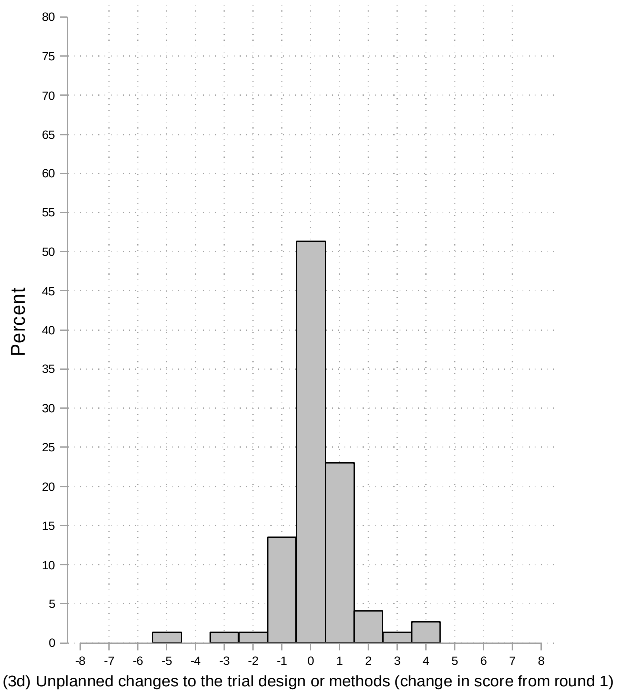

Additional file 9: Distributions of change in ratings from round 1 and Bland-Altman plots

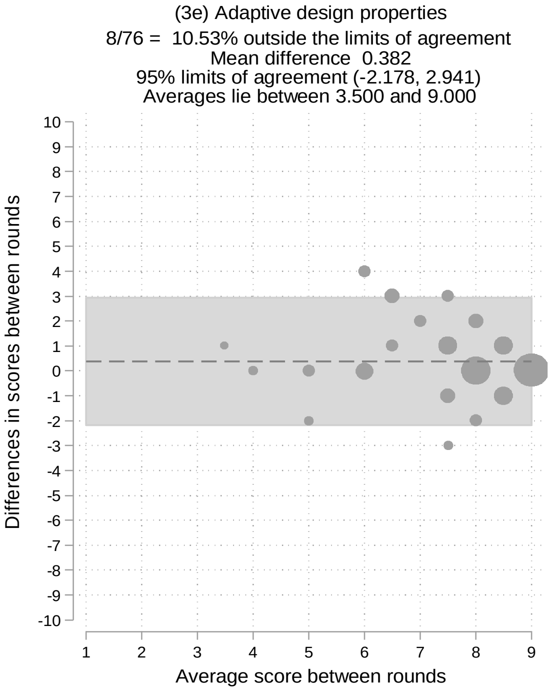

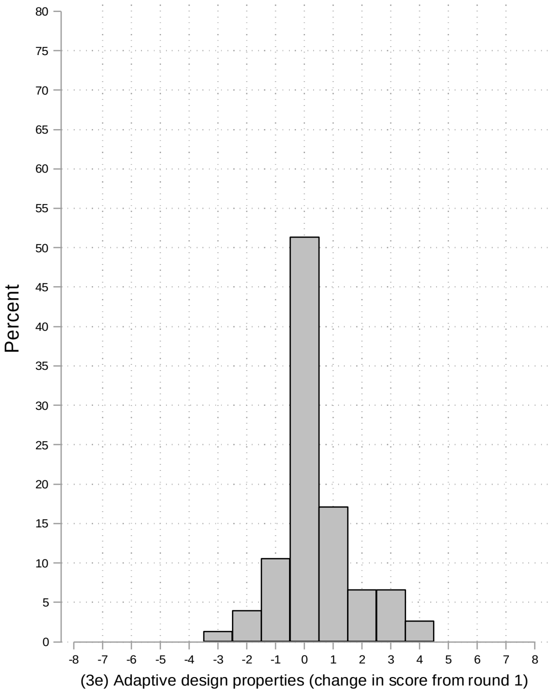

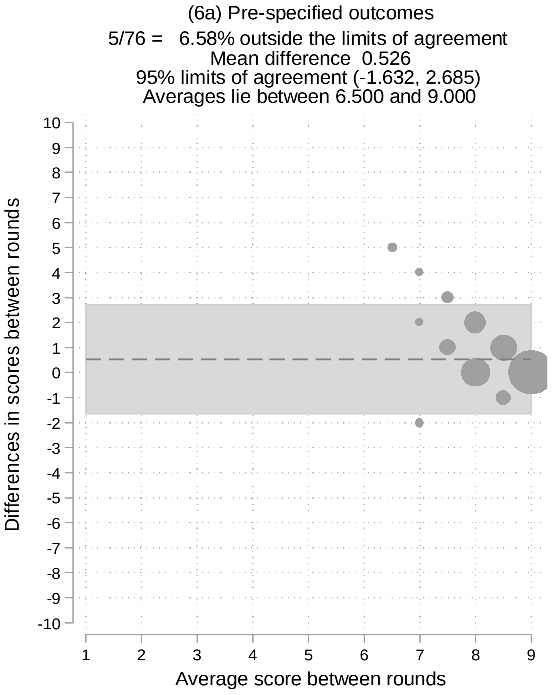

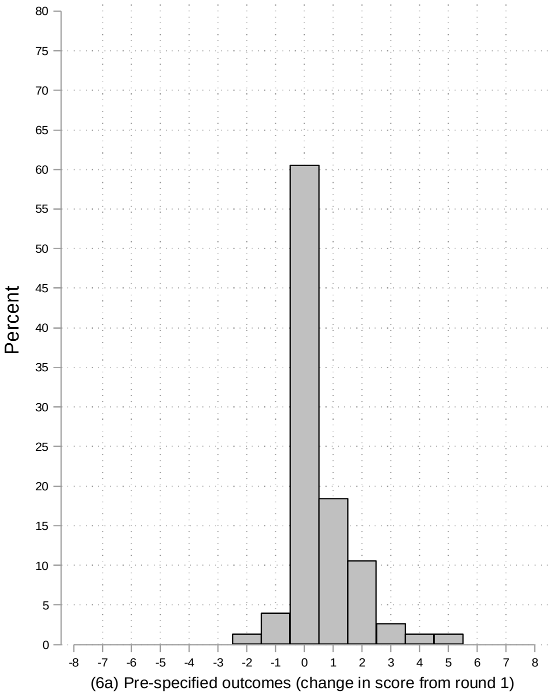

Additional file 9: Distributions of change in ratings from round 1 and Bland-Altman plots

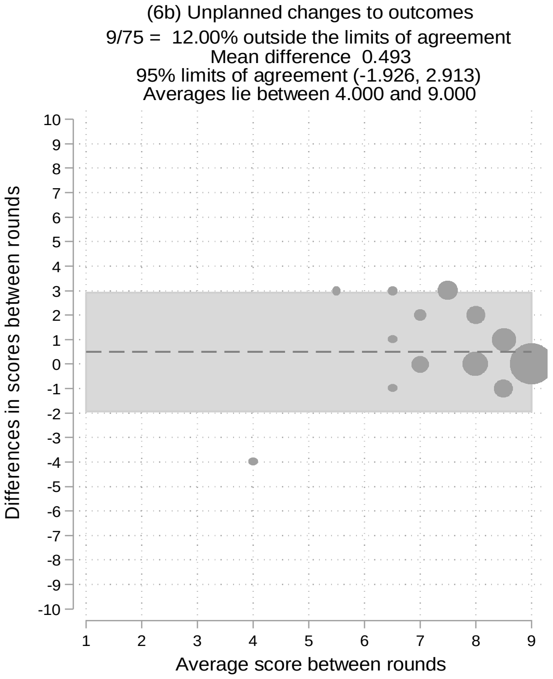

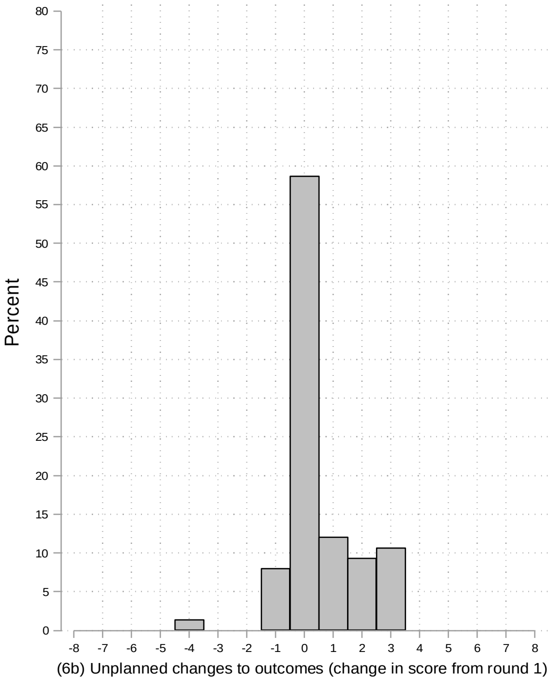

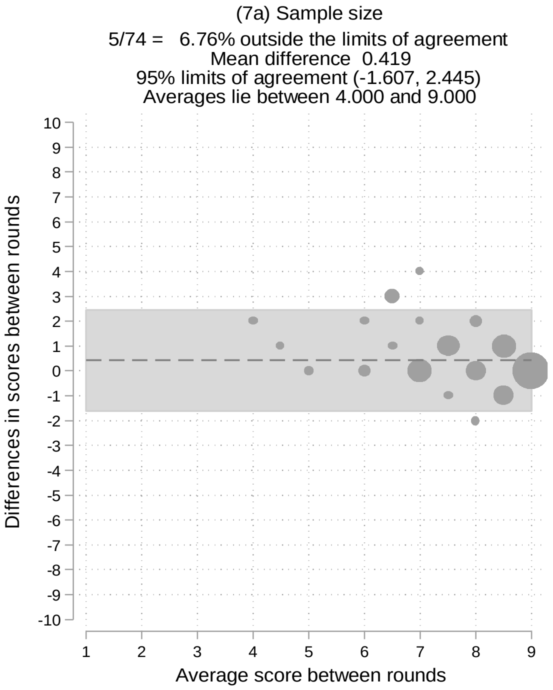

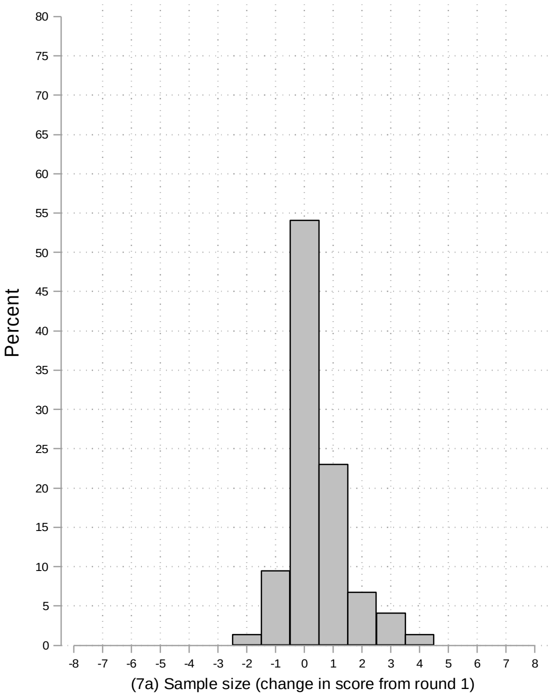

Additional file 9: Distributions of change in ratings from round 1 and Bland-Altman plots

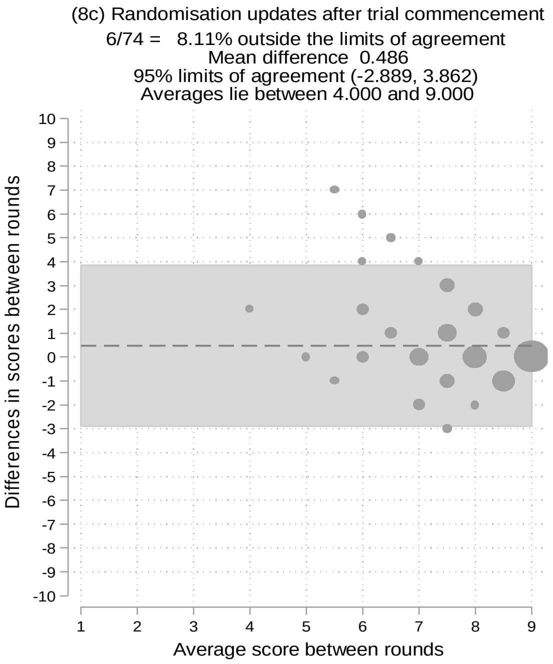

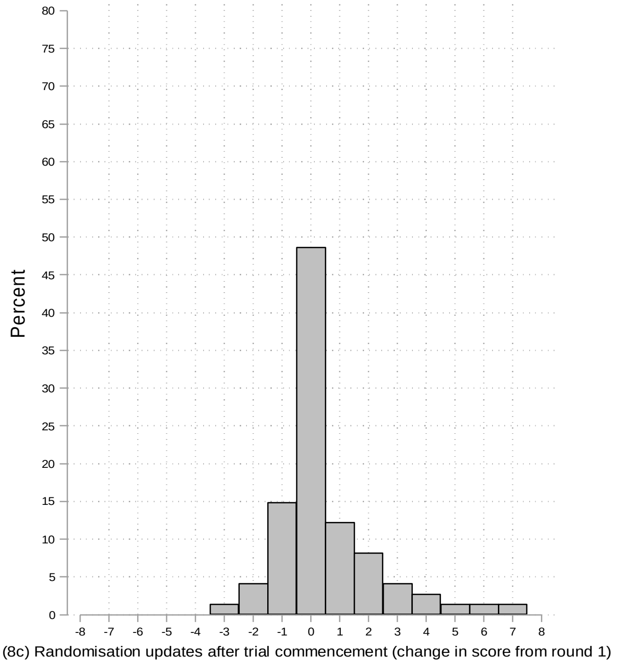

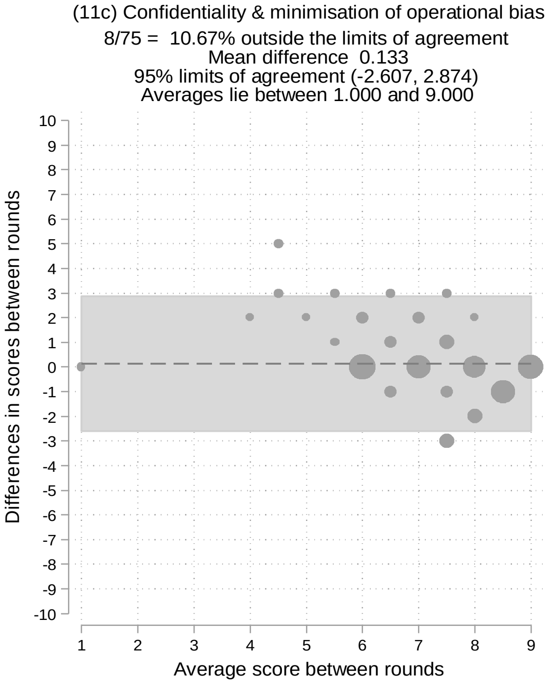

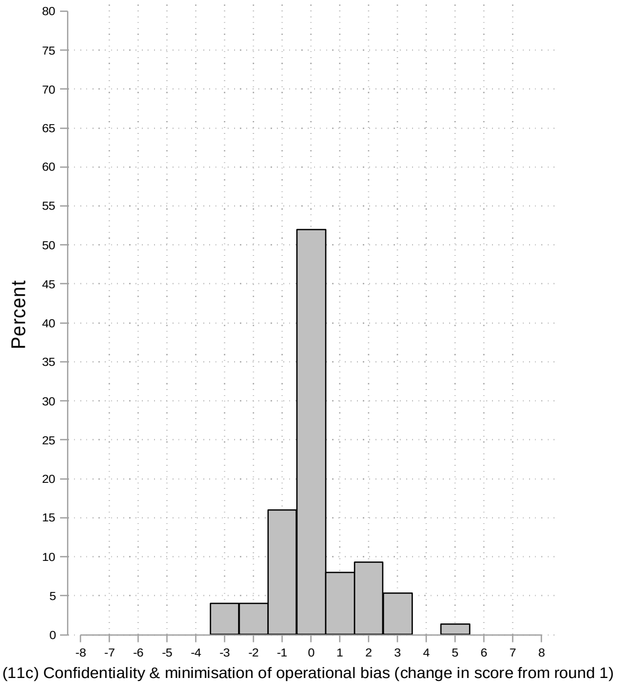

Additional file 9: Distributions of change in ratings from round 1 and Bland-Altman plots

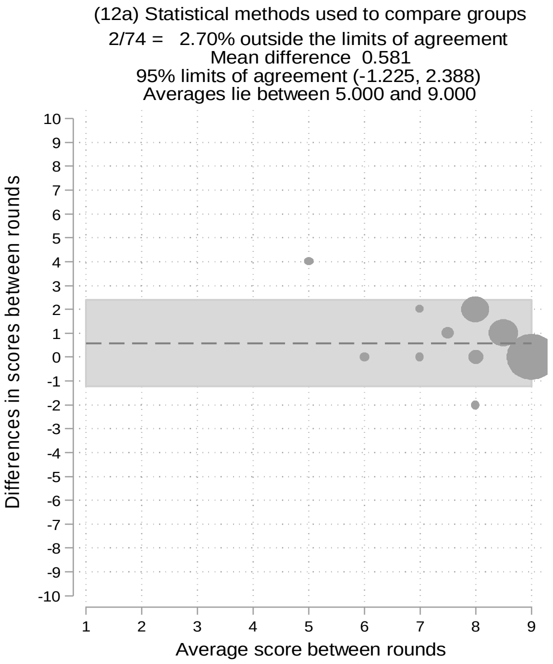

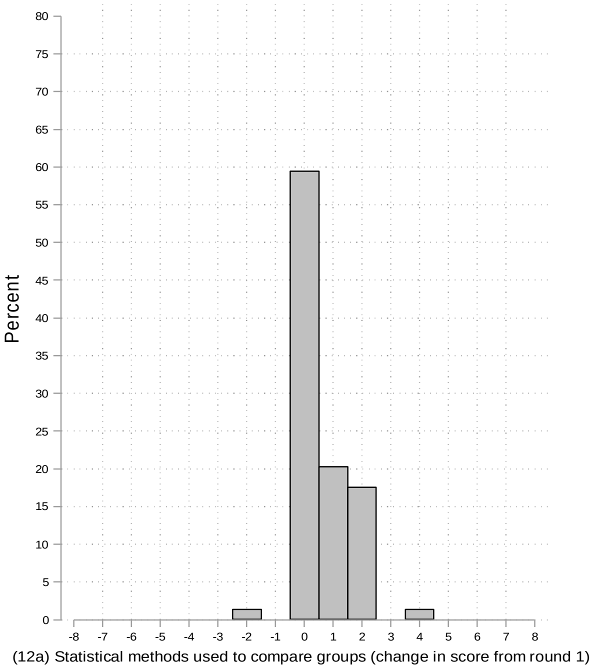

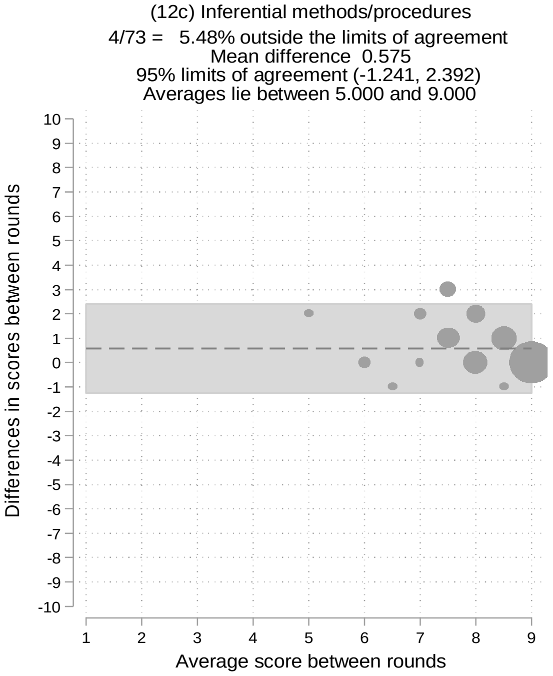

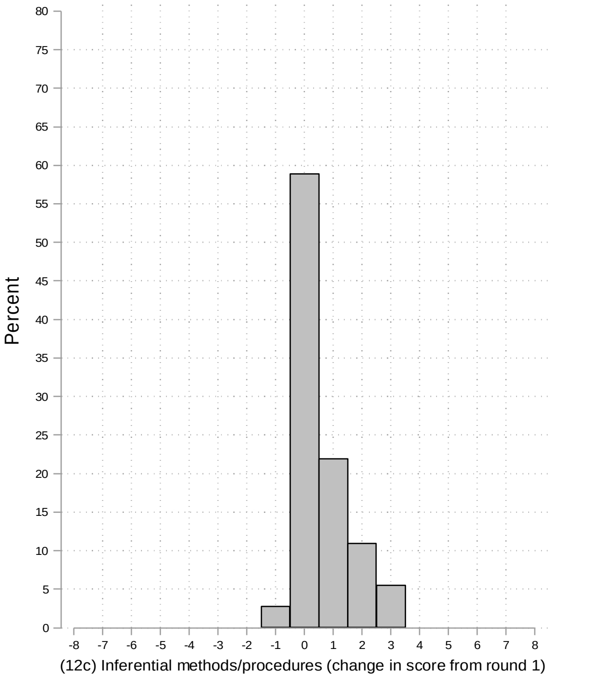

Additional file 9: Distributions of change in ratings from round 1 and Bland-Altman plots

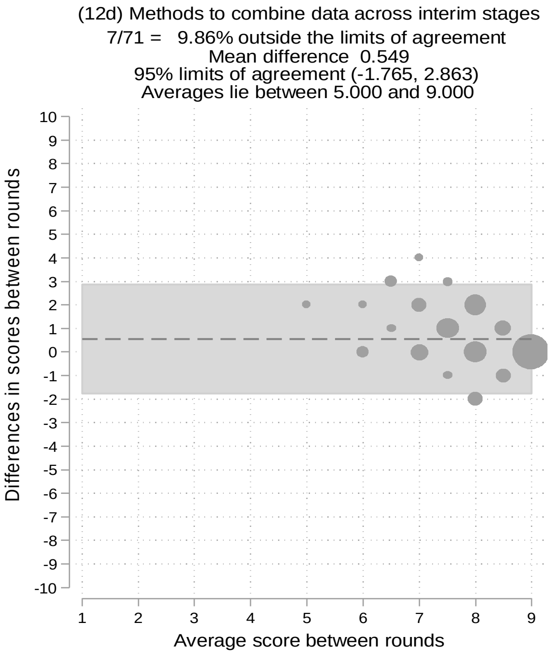

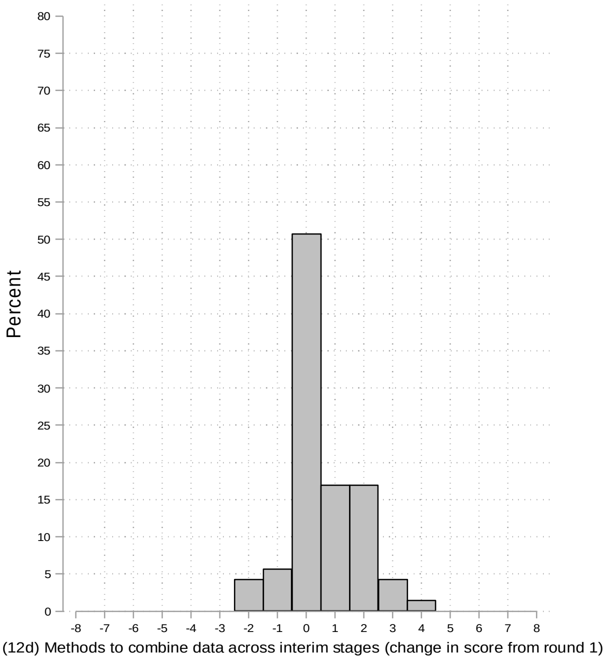

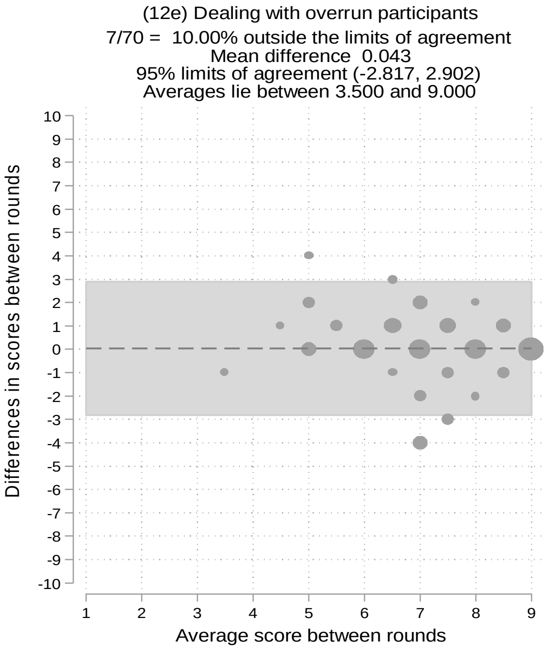

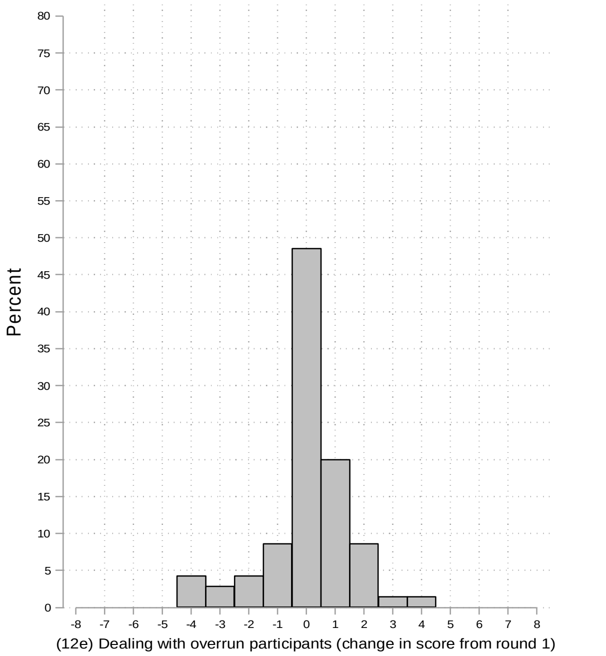

Additional file 9: Distributions of change in ratings from round 1 and Bland-Altman plots

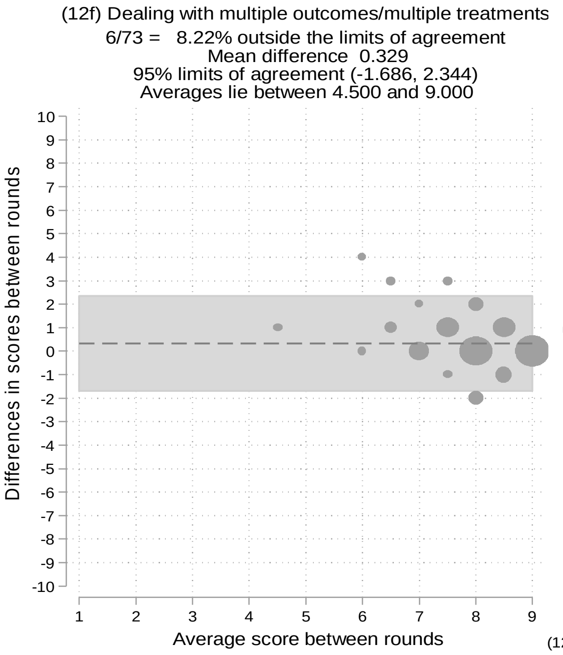

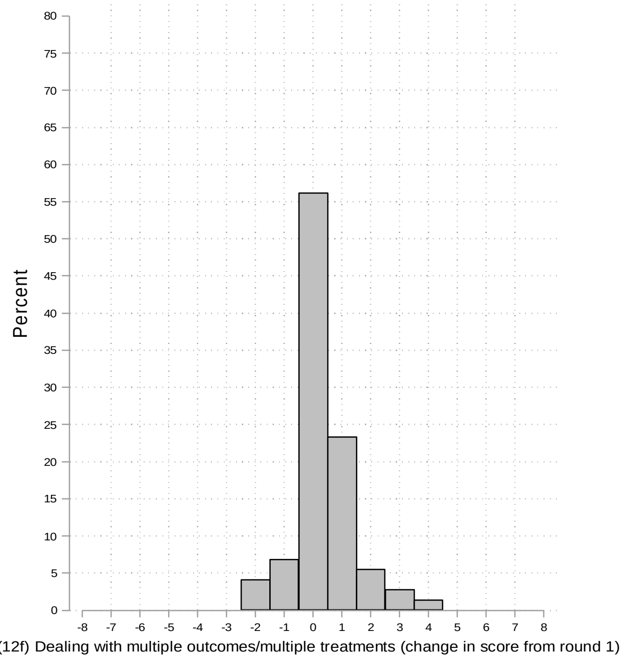

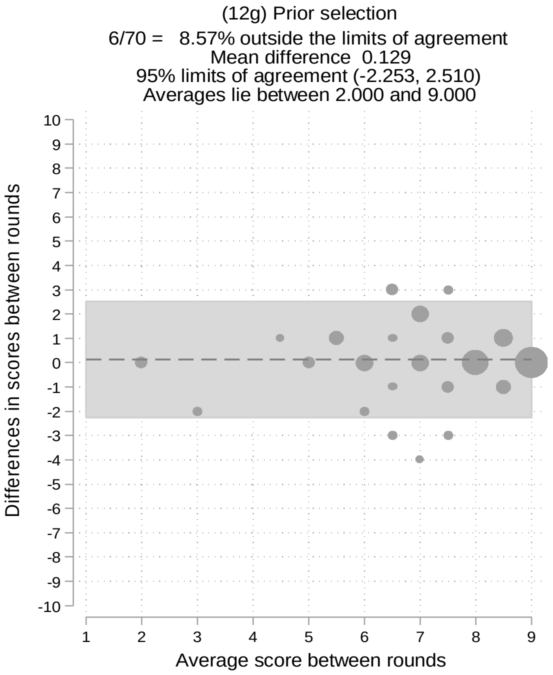

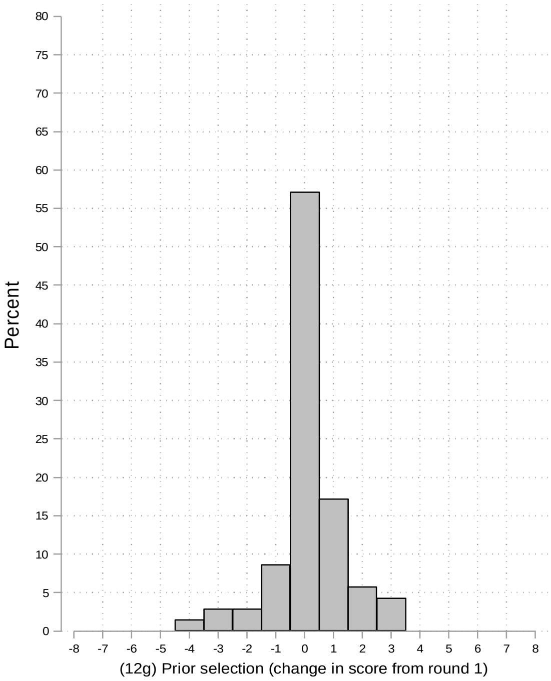

Additional file 9: Distributions of change in ratings from round 1 and Bland-Altman plots

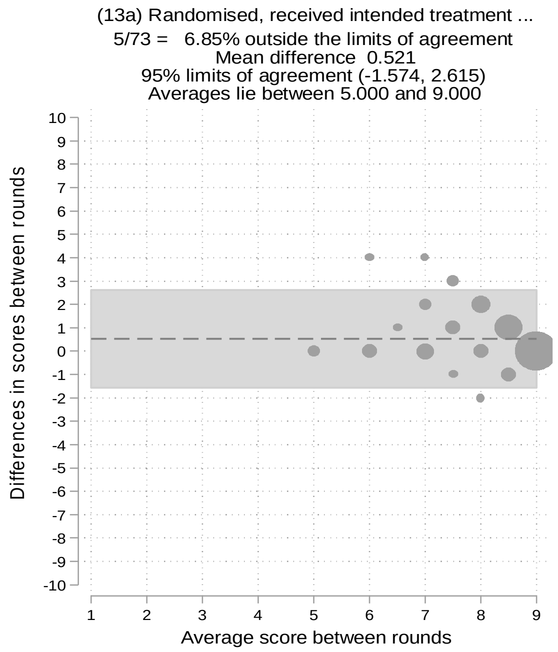

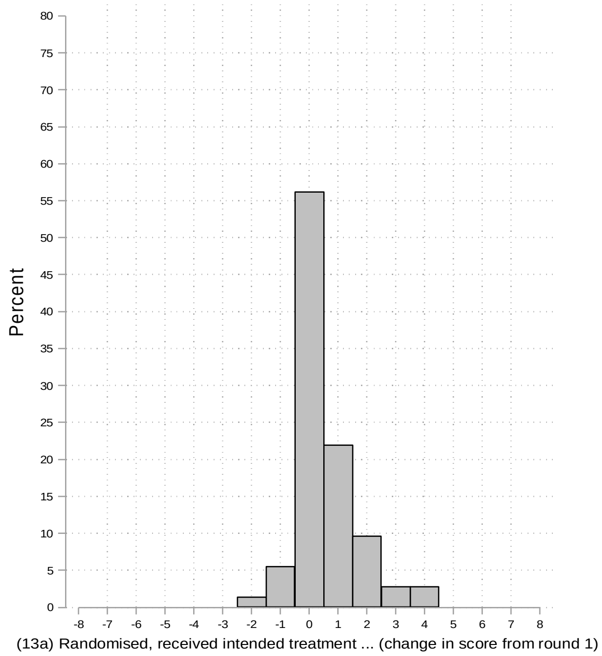

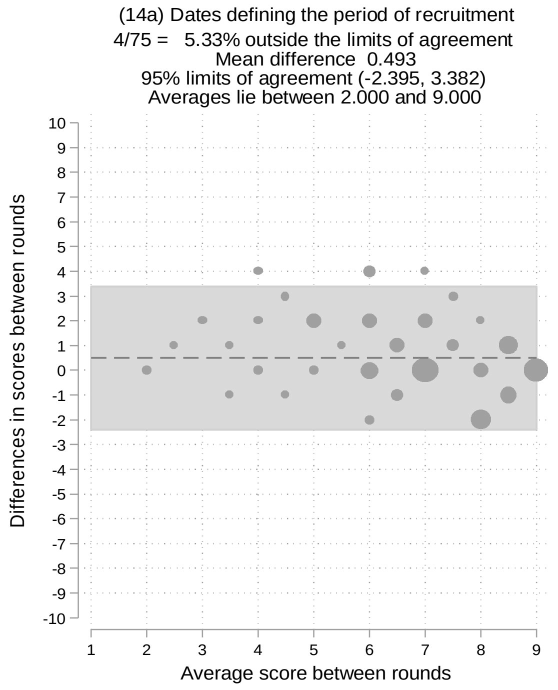

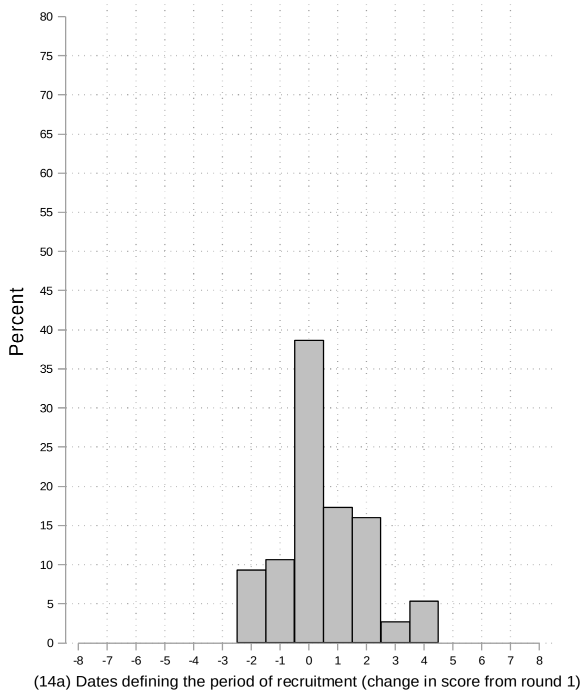

Additional file 9: Distributions of change in ratings from round 1 and Bland-Altman plots

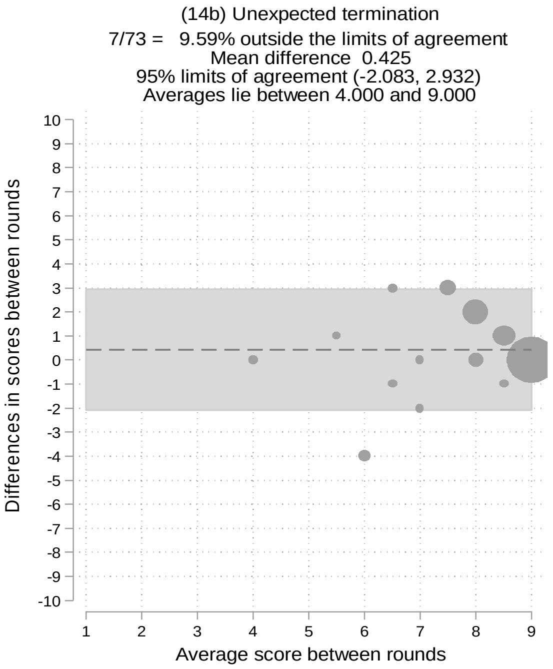

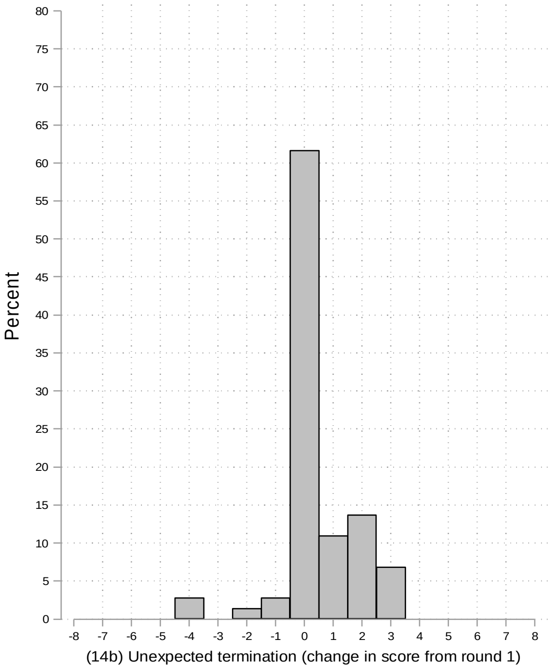

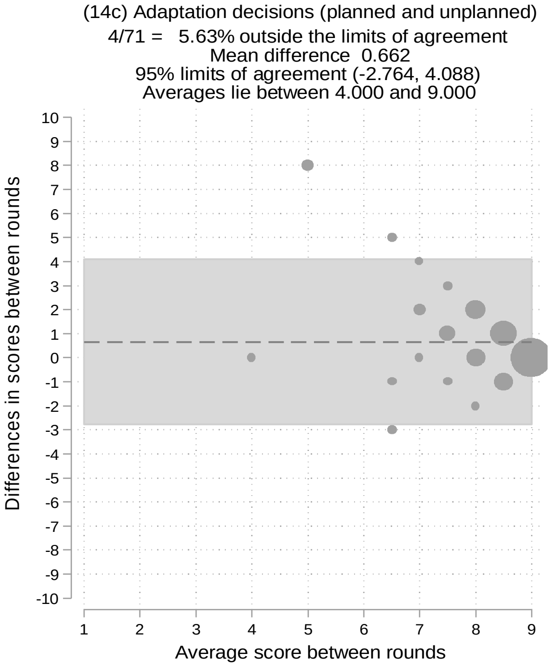

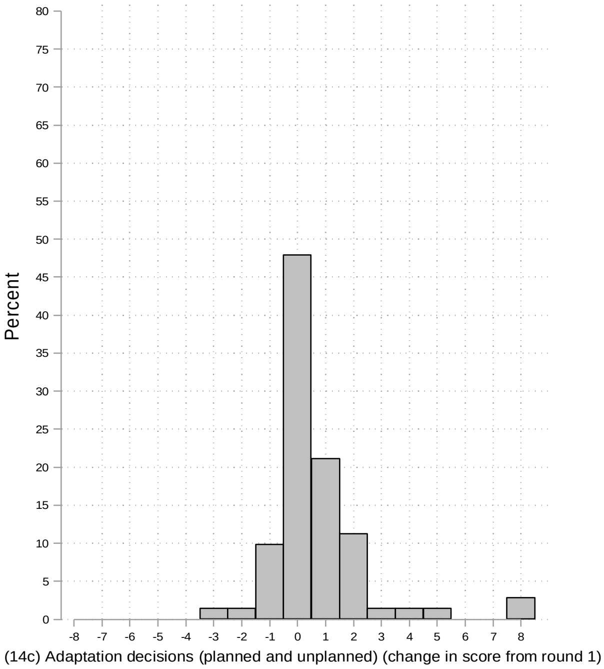

Additional file 9: Distributions of change in ratings from round 1 and Bland-Altman plots

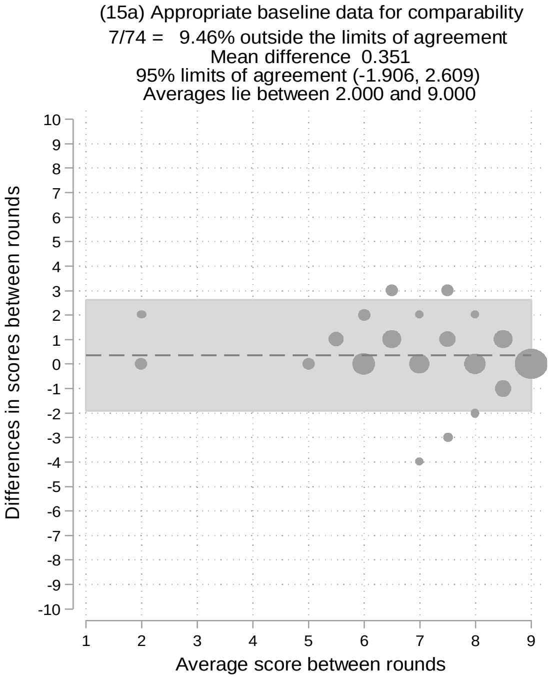

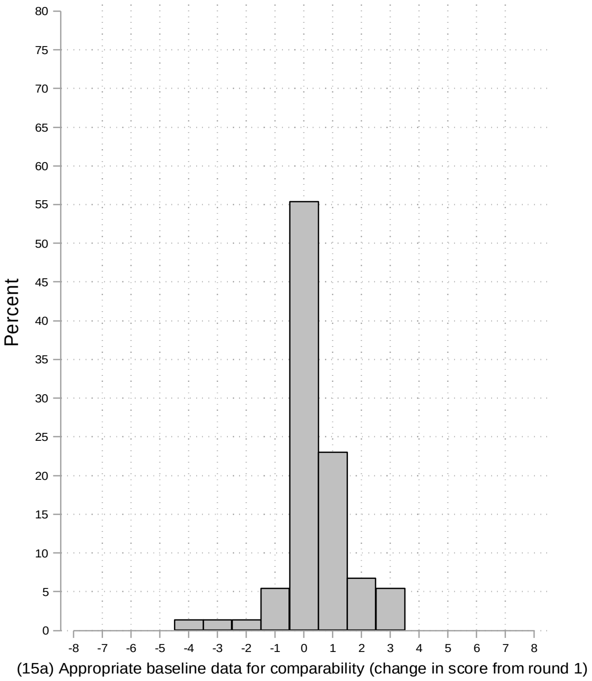

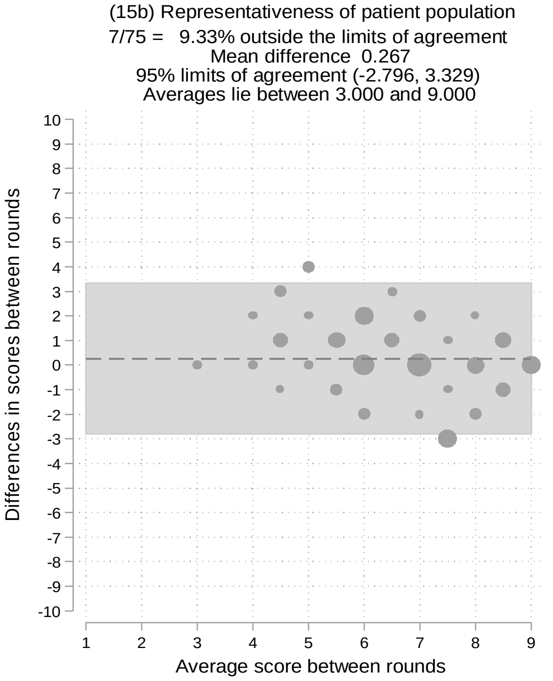

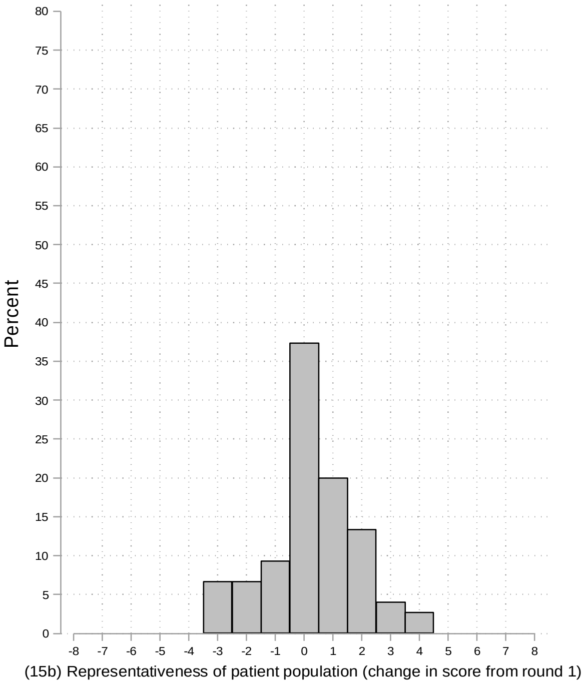

Additional file 9: Distributions of change in ratings from round 1 and Bland-Altman plots

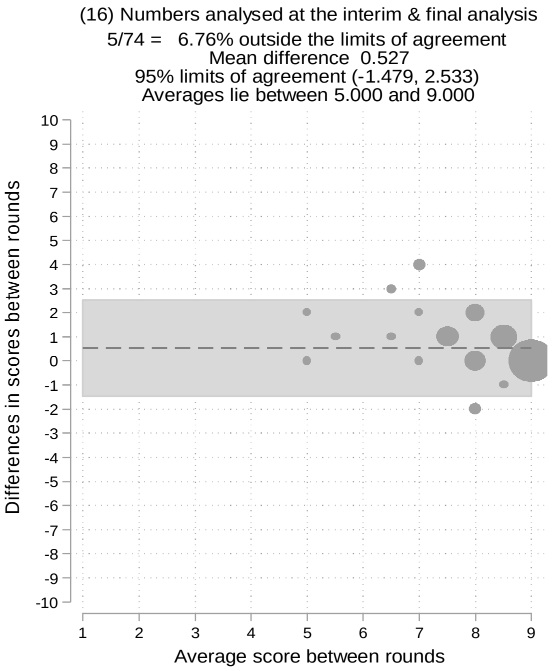

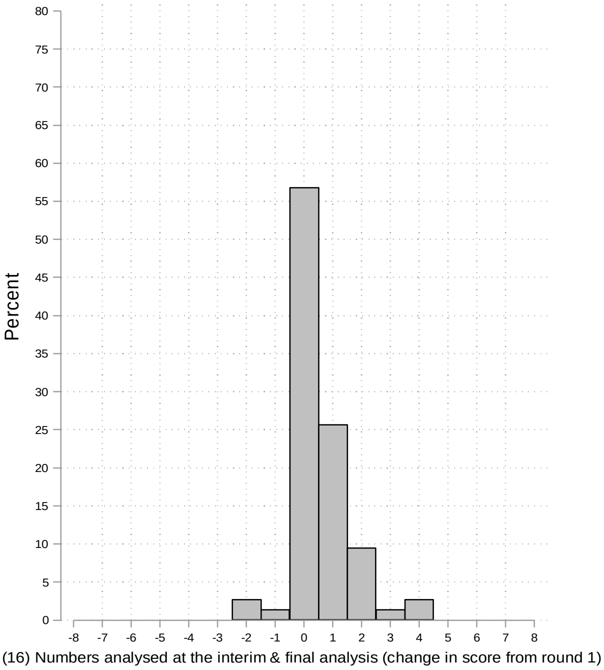

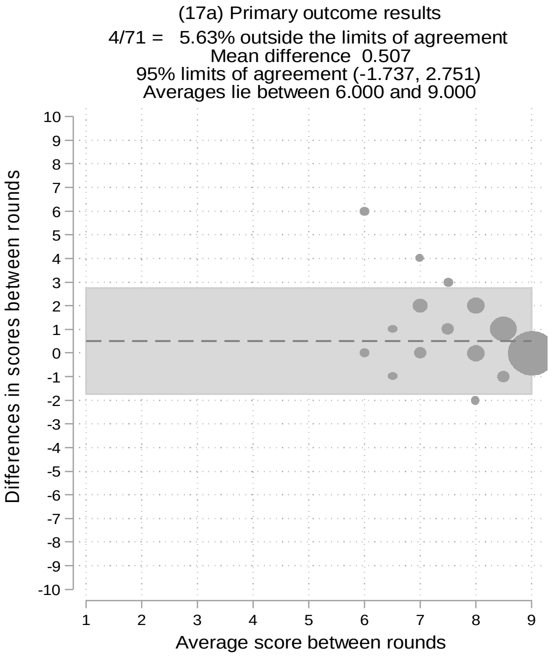

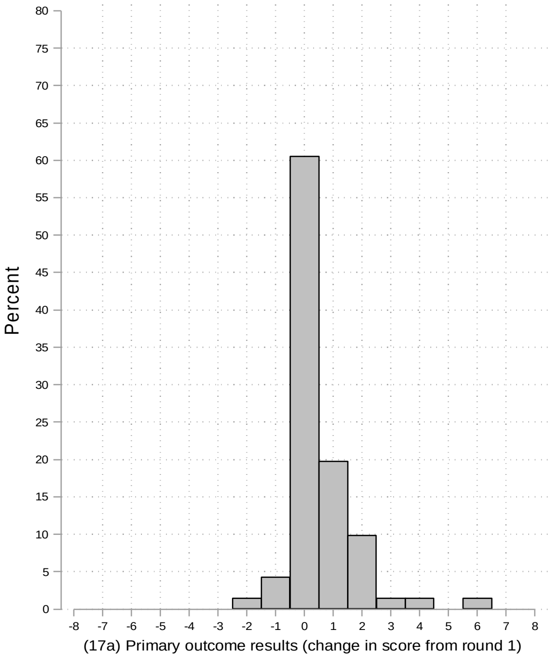

Additional file 9: Distributions of change in ratings from round 1 and Bland-Altman plots

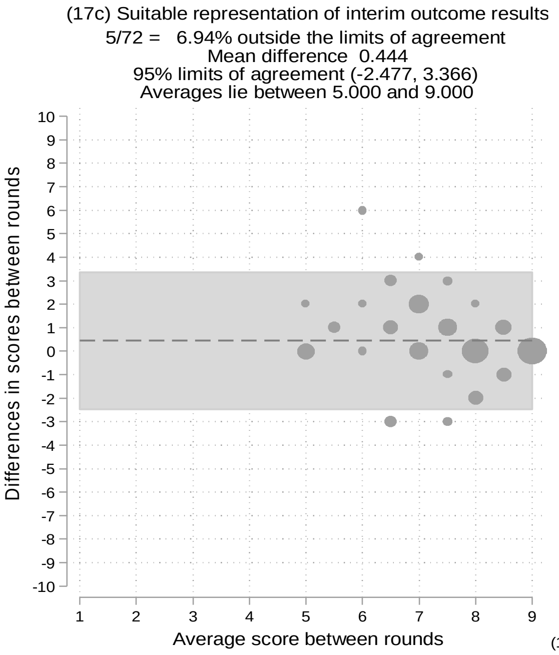

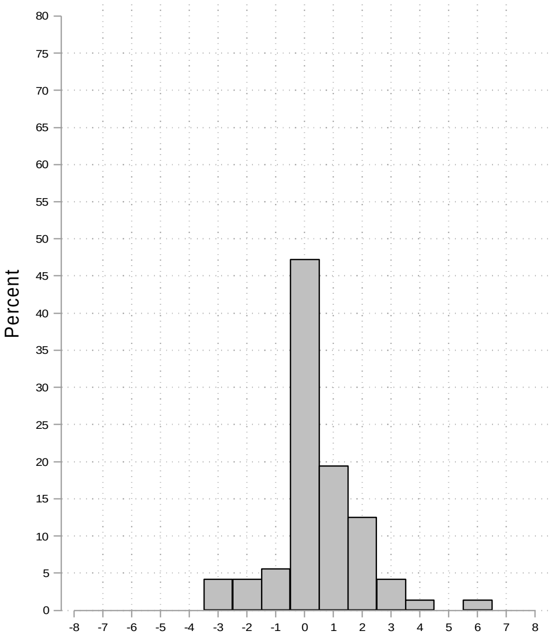

(17c) Suitable representation of interim outcome results (change in score from round 1)

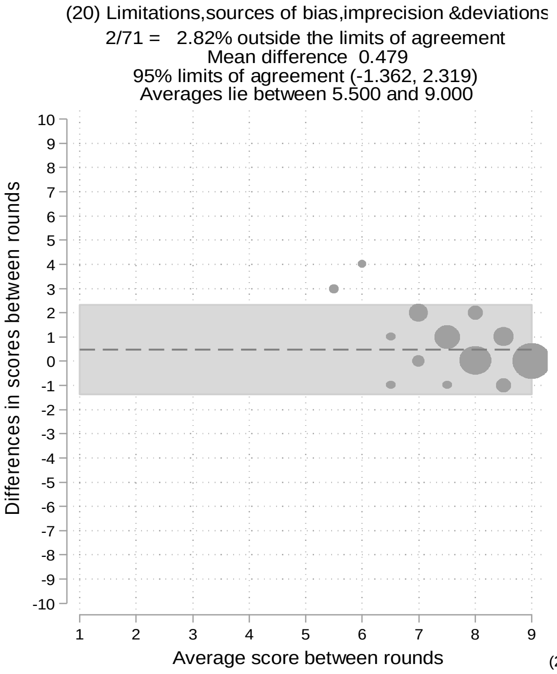

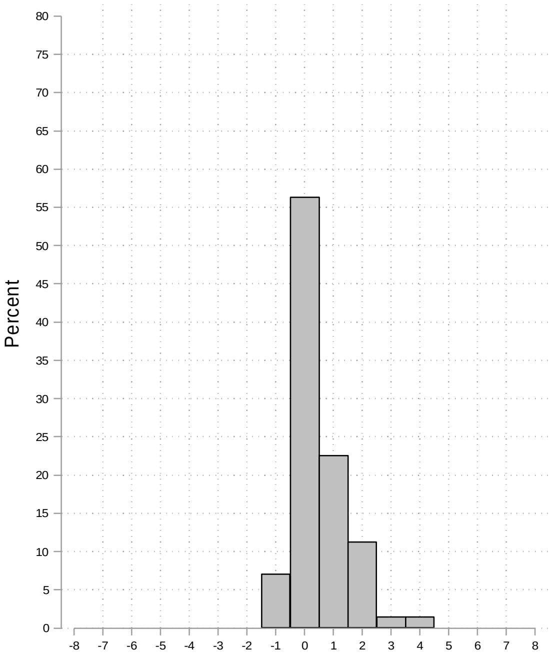

(20) Limitations,sources of bias,imprecision &deviations (change in score from round 1)

Additional file 9: Distributions of change in ratings from round 1 and Bland-Altman plots

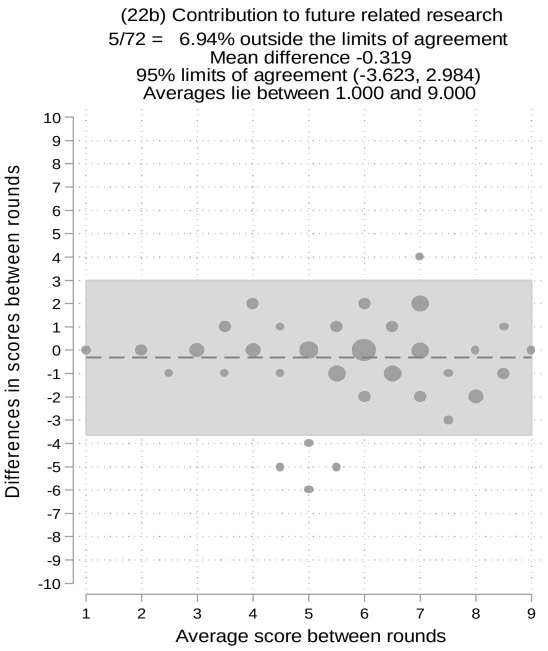

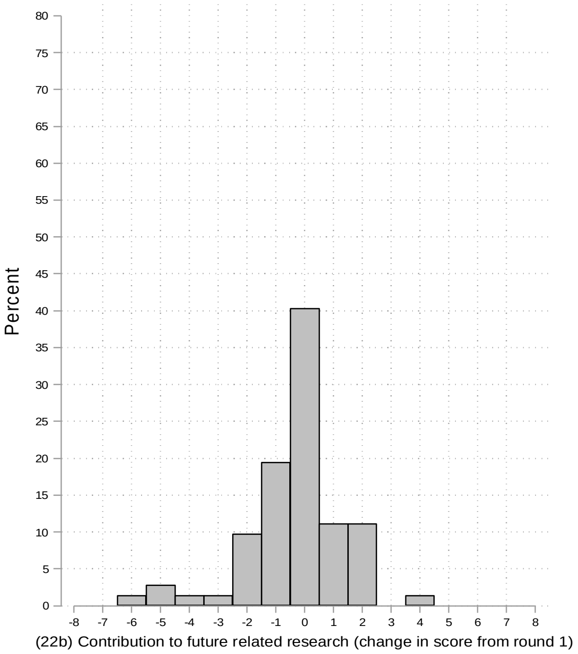

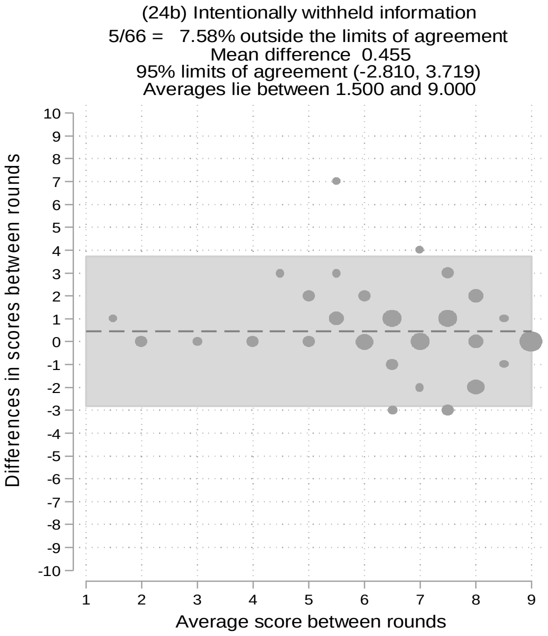

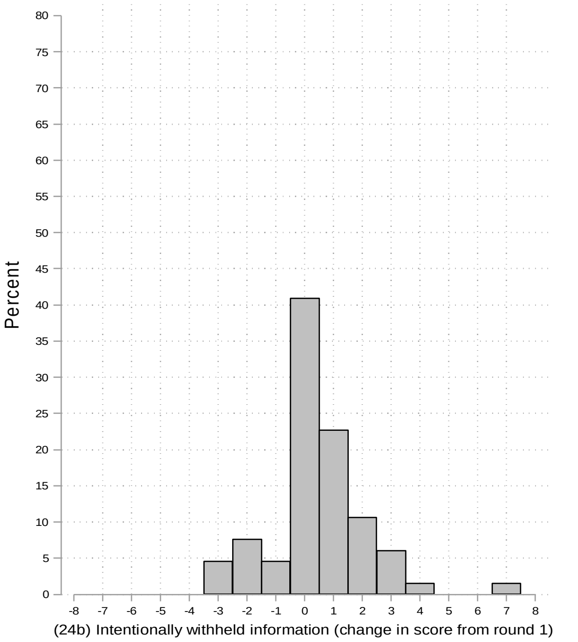

Additional file 9: Distributions of change in ratings from round 1 and Bland-Altman plots

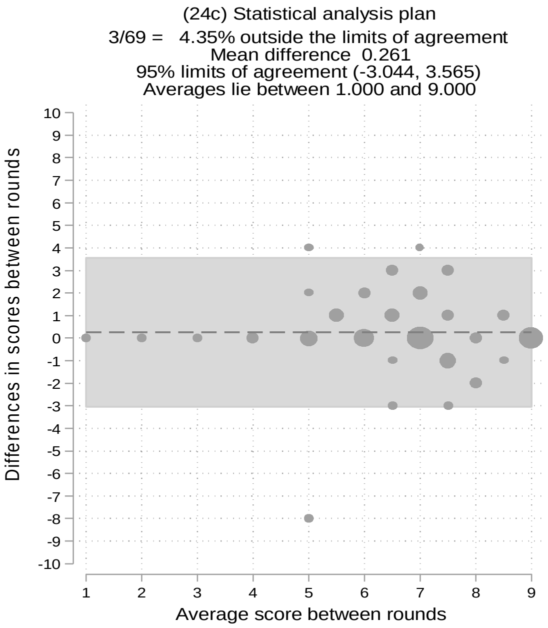

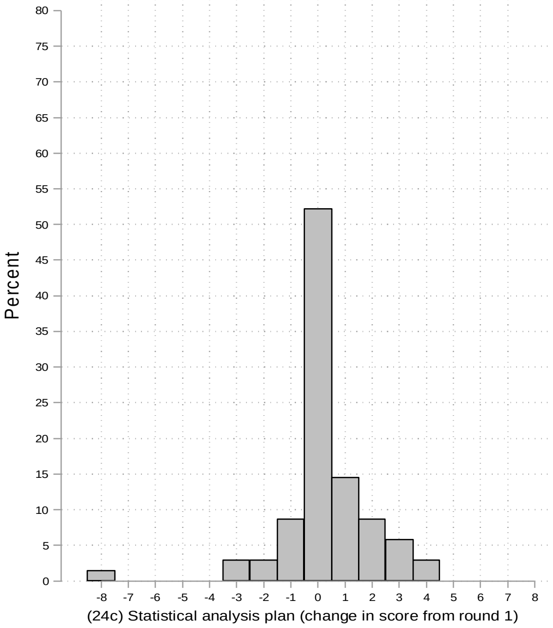

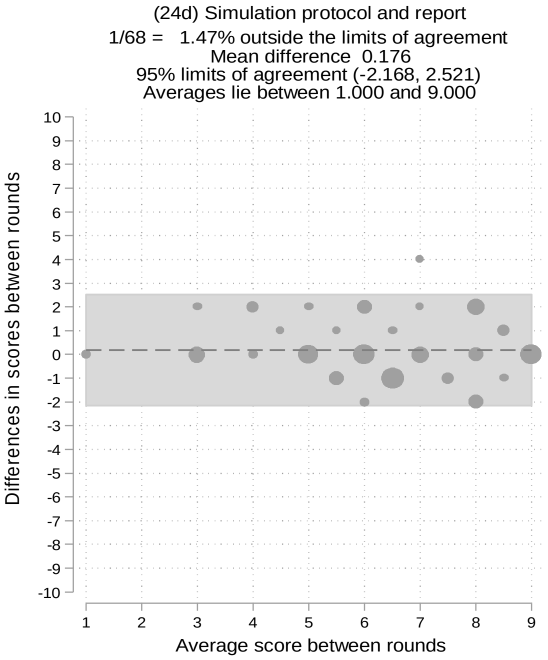

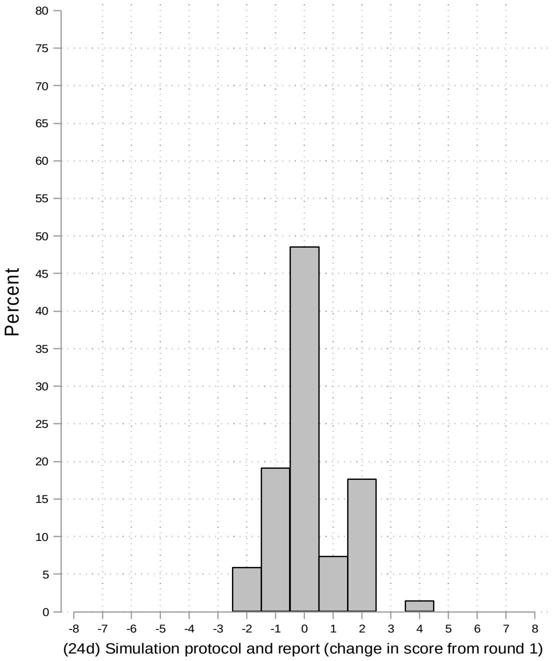

Additional file 9: Distributions of change in ratings from round 1 and Bland-Altman plots

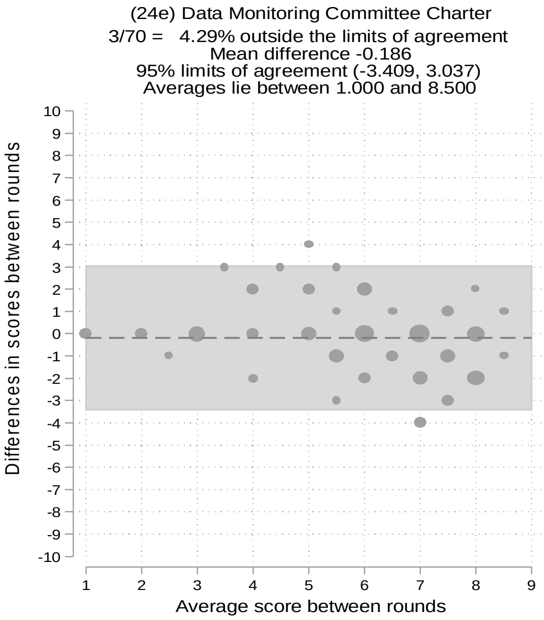

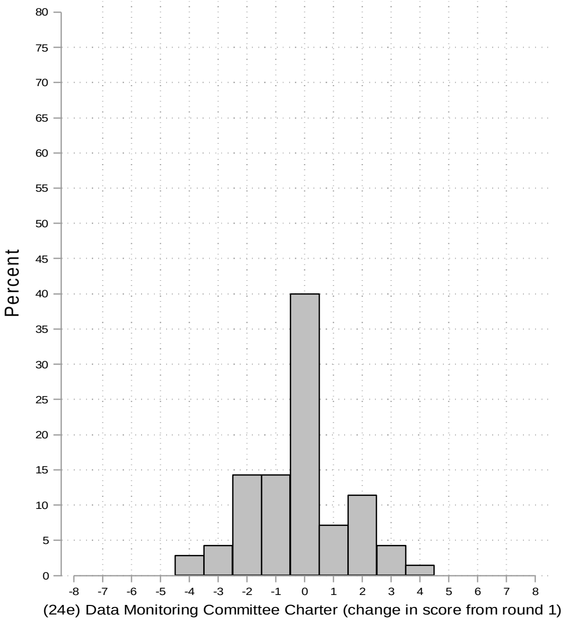

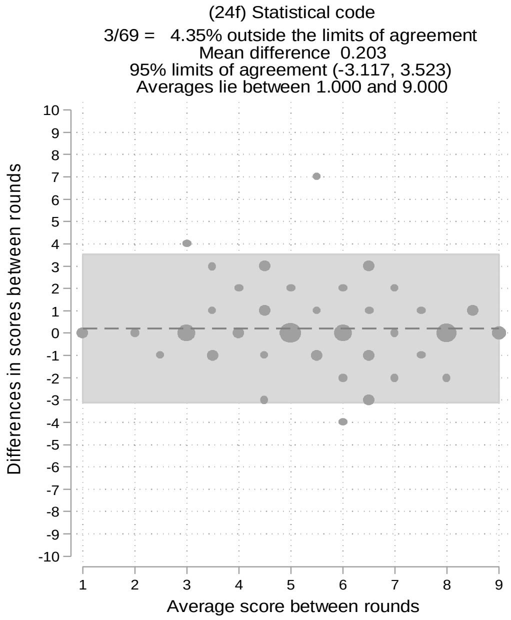

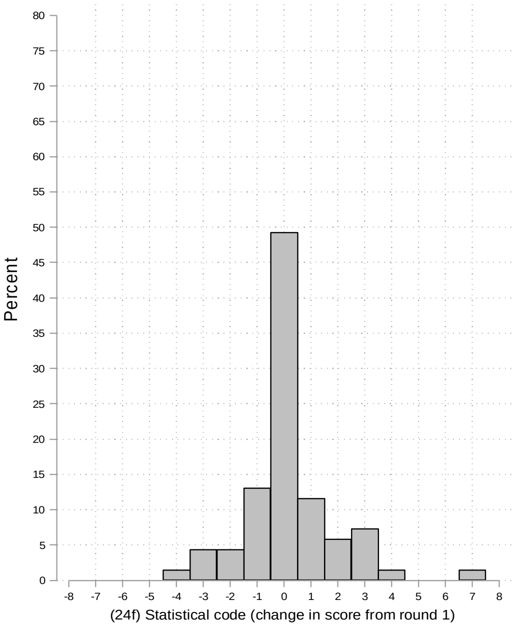

Supplement: Supplementary file 11 [file 12916_2018_1196_MOESM9_ESM.docx]
